# A Review of Indian-Based Drones in the Agriculture Sector: Issues, Challenges, and Solutions

**DOI:** 10.3390/s25154876

**Published:** 2025-08-07

**Authors:** Ranjit Singh, Saurabh Singh

**Affiliations:** 1 Department of Human and Digital Interface, Woosong University, Daejeon 34606, Republic of Korea; 202112053@live.wsu.ac.kr; 2 Department of AI & Big Data, Woosong University, Daejeon 34606, Republic of Korea

**Keywords:** drone technology, crop monitoring, soil analysis, training programs, technological adoption, farming innovations, UAV

## Abstract

In the current era, Indian agriculture faces a significant demand for increased food production, which has led to the integration of advanced technologies to enhance efficiency and productivity. Drones have emerged as transformative tools for enhancing precision agriculture, reducing costs, and improving sustainability. This study provides a comprehensive review of drone adoption in Indian agriculture by examining its effects on precision farming, crop monitoring, and pesticide application. This research evaluates technological advancements, regulatory frameworks, infrastructure, farmers’ perceptions, and the financial accessibility of drone technology in the Indian agricultural context. Key findings indicate that, while drone adoption enhances efficiency and sustainability, challenges such as high costs, lack of training, and regulatory barriers hinder widespread implementation. This paper also explores the growing market for agricultural drones in India, highlighting key industry players and projected market growth. Furthermore, it addresses regional differences in adoption rates and emphasizes the increasing social acceptance of drones among Indian farmers. To bridge the gap between potential and practice, the study proposes several policy and institutional recommendations, including government-led financial incentives, training programs, and public–private partnerships to facilitate drone integration. Moreover, this review article also highlights technological advancements, such as AI and IoT, in agriculture. Finally, open issues and future research directions for drones are discussed.

## 1. Introduction

With the global population expected to reach 9.7 billion by 2050, the demand for food is projected to rise significantly [1]. India is the second-largest food producer worldwide [2] and relies heavily on agriculture, which is the primary livelihood for its rural population. However, this sector faces many challenges, including small landholdings, unpredictable weather, low productivity, and dependence on traditional farming methods. To elaborate, the agricultural industry has been slow to adopt the latest technological advancements. The main obstacles include crop loss caused by unpredictable weather and uncontrolled pest attacks. Many Indian farmers still depend on seasonal monsoon rains for irrigation and continue using traditional farming practices. Additionally, Indian agriculture faces other issues, such as structural hurdles that lead to low productivity, like minimal landholdings that are often not profitable [3]. The average farm size in India is much smaller (around 1.08 hectares) compared to countries like the USA, Australia, and European nations [4]. Despite having smaller farms, many Indian farmers have limited access to essential inputs such as water, fertilizers, and electricity, mainly due to economic and infrastructural constraints. Furthermore, there is minimal automation, including the use of tractors, harvesters, drip irrigation, and similar technologies. Indian agricultural yields are among the lowest globally, due to factors like low soil fertility, insufficient fertilizer use, and traditional farming practices. Additional challenges include soil degradation, overuse of leguminous and fodder crops, and unscientific farming methods, all of which increase the difficulties faced by farmers [5].

**Figure 1:**
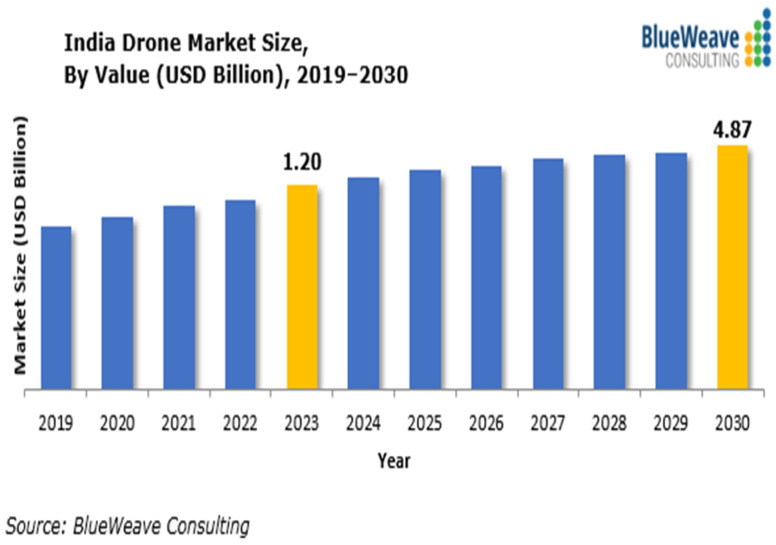
Projected growth of the Indian drone market [7].

The government of India has set an ambitious goal to double farmers’ income in the coming years [6], creating an urgent need for improvements in this field. The agricultural sector must begin adopting precision agriculture to enhance farmers’ productivity. To overcome these challenges, Indian agriculture needs to embrace advanced technology. Among these innovations, drones have become transformative tools that offer precision agriculture capabilities, lower costs, and support environmental sustainability. As shown in Figure 1, the Indian drone market, which includes both commercial and non-commercial applications like agriculture, defense, healthcare, infrastructure, and surveillance, is expected to grow from USD 1.2 billion in 2023 to USD 4.87 billion by 2030, at a compound annual growth rate (CAGR) of 22.15% [7]. This rapid expansion is fueled by increasing demand for drones in precision farming, land surveys, and disaster response, along with strong government support through initiatives like the Production-Linked Incentive (PLI) scheme for drone manufacturing. Although this projection encompasses the entire UAV market in India, agriculture remains a key area of growth due to its potential to reduce labor costs and boost efficiency in farm management.

Drones enable farmers to increase crop yields and promote environmental sustainability through precision agriculture and innovative farming methods. They help cut costs by reducing manual pesticide spraying expenses and lowering pesticide exposure for workers [8]. Drones allow for efficient and fast pesticide application, improving pest control and decreasing environmental pollution. India’s diverse agro-climatic zones result in varied cropping patterns and agricultural practices [9].

This diversity necessitates specialized solutions for crop monitoring and management, making drones an effective tool for precision agriculture across various regions. Additionally, drones are equipped with sensors that enable crop health monitoring throughout the growing season, allowing farmers to make timely interventions based on assessments of nutrient deficiencies and pest attacks. With increasing water scarcity in many Indian regions, efficient water management becomes essential [10]. Drones can help monitor soil moisture levels and crop health, enabling farmers to optimize irrigation practices and conserve water resources.

Additionally, pest and weed prevalence continue to challenge Indian agriculture [11]. Drones serve as an early warning system for disease detection, weed management, and the identification of issues before visible symptoms appear. Furthermore, drones provide real-time, high-quality aerial imagery that surpasses satellite imagery and helps assess soil properties. The use of drones ensures need-based, precise, and focused applications for crop inputs, such as determining the correct amounts of fertilizers and pesticides to use, identifying areas for irrigation, assessing production readiness, and locating areas for planting and harvesting. This can be performed in real time using minimal resources.

Beyond current use cases, drones are expected to play a crucial role in the transition to Agriculture 4.0, which emphasizes integrating the Internet of Things (IoT), big data, artificial intelligence, and robotics throughout the entire agricultural production and supply chain [12].

Global trends show promising results. Countries like the United States and Japan have demonstrated how drones can boost agricultural productivity. However, India’s specific challenges, such as small farm sizes and limited access to technology, call for customized solutions suited to its socioeconomic and agro-climatic conditions.

Recent research by Trappery et al. in the field of agricultural drones discussed the specifications, advantages, and disadvantages of drones in agriculture [13]. This study offers valuable insights into technological advancements, operational capabilities, broad benefits, and limitations of drone usage, highlighting state-of-the-art developments and future R&D trends in agricultural UAV technologies. However, it does not sufficiently address the specific needs of small-scale farmers worldwide, leaving a gap in understanding how drones can be effectively adapted to different socioeconomic contexts.

Additionally, several research studies specific to the Indian agricultural context, such as drone benefits, economic aspects, and regulatory policies, were selected and analyzed, as shown in Table 1. These studies offer valuable insights into the advantages of drones across various sectors (Gupta et al., Katekar et al., Puppala et al., Ramanjaneyulu et al.), economic impacts (Katekar et al., Puppala et al., Ramanjaneyulu et al.), infrastructure development (Katekar et al., Puppala et al., Goyal et al.), and more. Among these, Puppala et al. stand out by providing a detailed categorization and ranking of barriers that hinder drone adoption among Indian small-scale farmers. Their study identifies six major types of barriers: economic, operational, regulatory, social, behavioral, and infrastructural, based on a hybrid multi-criteria framework combining Fuzzy Delphi and AHP methods. The findings emphasize that high initial investment and component costs are the most critical issues, followed by a lack of skilled operators, unclear policies, limited-service infrastructure, and insufficient awareness. Concerns over automation-related job displacement and region-specific challenges like weather and no-fly zones were found to be moderately significant. These results are validated by experts and supported by existing literature, reinforcing the urgency of addressing economic and operational gaps to ensure successful drone integration in Indian agriculture. Similarly, Yadav et al. discussed the current state of agricultural drone technology, including crop health monitoring and farm activities such as weed control, evapotranspiration estimation, and spraying [14].

Although these points are essential, their discussion does not thoroughly explore the challenges. However, these studies often lack comprehensive practical insights into the economic feasibility for smallholders. Furthermore, most studies do not consider training and certification, which are necessary for farmers and drone operators, since flying a drone requires specific skills [19]. Operational challenges such as battery life and payload capacity are also overlooked. Additionally, regulatory issues under current policies and the infrastructure for drone operations remain underexplored. This study aimed to address these gaps by providing an in-depth analysis of the economic, regulatory, and technological aspects of drone adoption in Indian agriculture. It assessed the opportunities and challenges of drone integration, especially for small-scale farmers. Moreover, this study features region-specific case studies from Punjab and Rajasthan to examine how drones are practically used in Indian farming. In doing so, it offers practical recommendations for policymakers, technology developers, and agricultural stakeholders to promote the scalability and long-term sustainability of drones in Indian agriculture.

**Figure 2:**
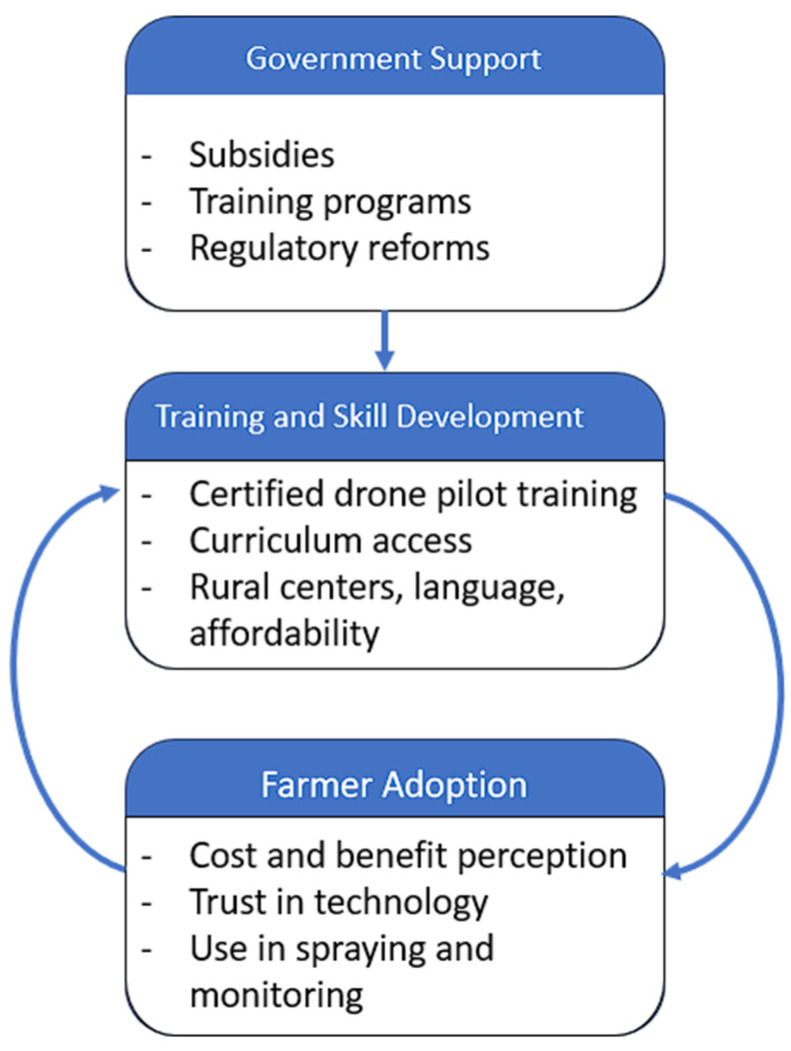
Conceptual framework illustrating the interaction between government policies, farmer adoption, and training in the agricultural drone ecosystem in India.

To frame these interconnected issues, a conceptual framework has been developed in Figure 2, illustrating the dynamic relationship between government support, training programs, and farmer adoption. This framework highlights how policy incentives and skill development initiatives influence the adoption of drone technology, and how farmers’ experiences can inform future policy and program improvements. It serves as a guiding structure for the review and analysis presented throughout this paper.

### 1.1. Contributions and Motivation

The research presented in this study explores the economic, regulatory, and technological progress related to the adoption of drone technology in Indian agriculture. It fills existing gaps in the literature concerning the feasibility and practical application of drones in farming. The key contributions of this study are as follows.

- This paper examines the widespread challenges faced by Indian farmers, based on the findings of numerous existing studies. It also highlights the main barriers to drone adoption in Indian agriculture, such as high costs, lack of technical training, regulatory obstacles, and poor infrastructure. This study provides valuable insights into the obstacles confronting small-scale farmers and the agricultural sector.
- The paper explores government initiatives and policies that promote drone use in agriculture, including financial assistance programs and regulatory reforms.
- The paper also explores various technological advancements, such as AI and machine learning integration, IoT-based monitoring, and solar-powered drones, along with policy solutions to tackle the identified challenges.
- This study explores real-world case studies and examines successful drone technology implementation in Indian agriculture, providing insights into cost-effective and scalable solutions.
- The survey offers insights into drones’ current and future market growth in India, along with the entities that are currently using drones for various purposes. It also highlights some open questions and research areas that can enhance drones’ capabilities.

The motivation behind this study is the urgent need to modernize Indian agriculture using advanced technologies such as drones. While several previous studies have highlighted the benefits of drones in agriculture, few have provided comprehensive insights into their economic feasibility, regulatory challenges, and practical implementation for smallholder farmers. Despite government initiatives aimed at promoting drone technology, policy gaps still exist that hinder small-scale farmers’ access.

This study not only highlights the slow adoption of technological advancements in farming and the structural hurdles that lead to low productivity but also offers existing solutions and bridges these gaps by providing an in-depth analysis of drone adoption in Indian agriculture.

### 1.2. Organization of Paper

The rest of the paper is structured as follows. Section 2 offers a thorough literature review, tracing the development of drones from military uses to agricultural applications. It discusses global trends in agricultural drones and their importance to India’s farming sector. Additionally, this section covers the policies and regulatory environment regarding drone use in India, emphasizing government initiatives and research efforts.

Section 3 explains the methodology applied in the study. It outlines the research approach, including the selection criteria for reviewed literature, data sources, and analytical techniques.

Section 4 provides an overview of the Indian agricultural drone landscape. It examines the current state of drone adoption in India, the main players in the agricultural drone industry, and the types of drones used in farming. This section discusses government initiatives, contributions from the private sector, and the economic impact of drones in agriculture.

Section 5 highlights the issues and challenges related to drone adoption in Indian agriculture. It covers financial constraints, regulatory hurdles, lack of farmer awareness, and insufficient infrastructure as key obstacles. Also, it looks at the economic viability of drones for small-scale farmers and emphasizes the need for effective training programs.

Section 6 examines technological and policy solutions to address the challenges identified earlier. It discusses advancements like AI and machine learning integration, IoT-based monitoring, and solar-powered drones for cost effectiveness. Additionally, it reviews policy recommendations such as government subsidies, training programs, and financial support initiatives to help small-scale farmers access drone technology. It also includes case studies of successful drone deployments in Indian agriculture.

Finally, Section 7 concludes the work by summarizing the key findings, emphasizing the transformative potential of drones in Indian agriculture. It highlights the importance of continued investment in drone technology, regulatory support, and farmers’ education to ensure the long-term success of drones in revolutionizing the agricultural sector. Additionally, it explores future research directions in drone technology for agriculture. Lastly, it concludes with an open discussion.

## 2. Literature Review

Drones have become transformative tools in precision agriculture, providing benefits such as better crop monitoring, targeted pesticide application, and increased productivity. This section reviews current research on agricultural drone adoption in India, emphasizing global advancements and their relevance to India’s farming sector. The main areas covered include technological innovations, economic viability, regulatory frameworks, and the challenges faced by small-scale farmers in India.

### 2.1. Drone Adoption in India

India operated its first drone in 1996 when the army acquired the Israeli Searcher Mark I. This marked the start of India’s engagement with unmanned aerial vehicles (UAVs) for military uses. Following the army’s lead, the Indian Air Force and Indian Navy also began to use drones in their operations within two years of the initial purchase. This early use mainly focused on gathering intelligence and reconnaissance. Over the years, India has advanced its drone technology, especially through efforts by organizations such as the Defense Research and Development Organization (DRDO) [20] and the National Aerospace Laboratories (NAL). The Rustom series of drones, including weapons-ready versions like Rust-II, is an example of domestic development aimed at replacing foreign models like the Israeli Heron [21]. The integration of drones into India’s national security efforts became more prominent, drawing lessons from global military experiences, particularly from countries like Israel.

Drones are now being used in agriculture for tasks like crop monitoring and pesticide spraying. Their use has gained significant attention worldwide and in India, where 70% of rural people are farmers. Since agriculture is a vital sector contributing 16% to India’s GDP, the government is actively working to implement this technology across various agricultural sectors.

### 2.2. Global Trends in Agricultural Drones

Unmanned aerial vehicles (UAVs), also known as drones, have revolutionized various sectors, and agriculture is one of them. Drones have evolved from simple, remote-controlled machines to performing complex tasks in agriculture autonomously. The agriculture sector worldwide has seen major changes in recent years due to the rapid adoption of drone technology. Globally, the use of drones in agriculture has enabled precision farming, helping farmers leverage data and technology for sustainable practices. Additionally, the ability to monitor and map larger areas in just a few hours makes this technology particularly valuable for farmers. Drones can produce accurate, up-to-date maps of farmland, providing farmers with essential information about their fields. Moreover, drones play a crucial role in irrigation management, which is vital in areas with limited water resources. They help optimize irrigation, reduce water waste, and ensure crops receive adequate water. Furthermore, drone technology has become increasingly common in planting and seed sowing. For example, in Australia, drones are increasingly used for reforestation efforts, especially in regions affected by annual bushfires. These unmanned aerial vehicles (UAVs) are equipped to disperse seeds across large, inaccessible terrains, aiding in rapid forest restoration [22]. A variety of imaging tools, such as thermal cameras, multispectral sensors, and light detection and ranging (LIDAR), can be mounted on drones to enable early detection of plant diseases. By capturing high-resolution aerial images, drones can identify subtle changes in crop health, allowing for timely intervention to prevent the spread of infections and reduce crop losses [23]. A comprehensive review indicates that as of December 2023, China and the USA have made significant contributions to research on UAV-based plant disease detection, with 25 and 18 research articles, respectively [24].

Developed nations have successfully adopted drone technology across various sectors; specifically, it has been reported that in Japan, drones conduct almost 90% of aerial crop dusting and spraying. The planning, design, and construction of rice irrigation systems, along with the implementation of irrigation scheduling, demonstrate the use of drones in precision agriculture in African countries such as Nigeria. The US government is also working on legislation that allows the use of drone technology in agriculture [25]. China has widely adopted drone technology in agriculture, especially in pesticide spraying. In 2021, more than 120,000 drones were used to spray pesticides over 175.5 million acres of farmland [26].

Along with the global adoption of drones, recent technological advancements have greatly improved drone capabilities across different industries. Key developments include the following.

#### 2.2.1. Multispectral and Hyperspectral Imaging

Multispectral and hyperspectral sensors on drones enable data collection at various wavelengths, allowing for a comprehensive analysis of environmental features. This method is especially useful in agriculture, as it helps with early disease detection and monitoring crop health. These sensors provide insights into plant physiology that are not easily visible to the human eye when examining wavelength bands [27].

#### 2.2.2. Artificial Intelligence (AI) and Machine Learning Integration

New technologies like AI and ML improve their skills. Right now, there is a widespread AI boom globally. AI is making drones better at spotting weeds and pests, analyzing crop data, and giving advice on what fertilizers to use. Combining AI with drones also helps cut down on chemical use and reduces resource waste.

#### 2.2.3. Thermal Imaging

Drones equipped with thermal cameras can detect temperature changes, which is vital for tasks like identifying crop water stress and finding people during search and rescue operations. This capability allows operation in various environments, including those with limited visibility.

#### 2.2.4. Enhanced Autonomy and Swarming Technology

AI advancements have enabled the development of drone swarms that can operate independently and collaboratively. These swarms can work together without human help to perform complex tasks such as surveillance, emergency response, and large-scale environmental monitoring [28]. Because of these developments, drone usage has grown in recent years. As a result, several new companies have entered the agricultural drone market, which offers a wide range of drones and services. This increased competition reduces the cost of agricultural drones and makes them more affordable for farmers [29].

### 2.3. Relevance of Drones in Indian Agriculture

In India, most farmers have small landholdings, and external factors such as weather, soil conditions, and temperature significantly influence farming. Agricultural drones enable farmers to adapt to specific environments and make informed decisions [30]. The adoption of drone technology in agriculture is transforming traditional farming methods, increasing efficiency, and encouraging sustainable resource use. Drones are used in various innovative ways, such as crop spraying, where they evenly distribute pesticides, fertilizers, and herbicides across fields. This approach not only reduces labor costs but also minimizes farmers’ exposure to hazardous chemicals [31]. Crop health monitoring, which utilizes high-resolution cameras and artificial intelligence, detects early signs of diseases, pest infestations, and water stress. For example, in cashew farming, AI-driven drones have achieved up to 95% accuracy in identifying anthracnose disease in leaves, enabling timely treatment and decreasing crop loss [32]. Drones in India are also assisting with seed planting, especially in areas that are hard to reach or have difficult terrain [33].

Along with the current application of drones in agriculture, the Indian government has launched several initiatives to promote and regulate drone use. Key programs and regulations include the following.

#### 2.3.1. Digital Sky Platform

Launched by the Directorate General of Civil Aviation (DGCA), the Digital Sky Platform is a single-window online system for drone registration and approval processes in India. It offers an interactive airspace map that categorizes regions into green, yellow, and red zones to assist drone operations. Operators can register their drones, apply for necessary permissions, and obtain Remote Pilot Certificates through this platform [34].

#### 2.3.2. Kisan Drones

The “Kisan Drones” program was launched to modernize the agriculture industry and promote the use of drones for various farming tasks. These drones assist with activities like assessing crops and digitizing land records by applying fertilizers and insecticides. The program aims to advance precision agriculture, reduce labor costs, and improve efficiency [35].

### 2.4. Rules and Regulations

Every country has laws and regulations about flying drones in specific areas to prevent damage and collisions with property, and these laws differ from country to country. India also has laws and policies that govern drones. According to these policies, the regulatory authority, the Directorate General of Civil Aviation (DGCA), has classified drones into five main types, which are listed in Table 2 [36].

Based on the above classification, drone policies are regulated. India first implemented its policy in 2018. According to this policy, the rules and regulations for drones are outlined as follows.

#### 2.4.1. Old Policy 2018

- Pre-Flight Requirement

- Drones larger than the Nano category require a Unique Identification Number (UIN) from the aviation regulator, as shown in Table 3;
- Like vehicle registration, UIN incurred a fee of INR 1000 and was not issued to foreign entities;
- Operators of larger drones needed a Unique Air Operator permit (UAOP), like a driver’s license, costing INR 25,000 with a validity of five years [37].

b. Flying Conditions

- All drones, except Nano ones, were subject to mandatory equipment requirements, including GPS, anti-collision lights, ID plates, RFID, and SIM facilities;
- Software ensuring ‘no-permission, no takeoff’ was mandatory;
- Operators of small or larger drones need to file a flight plan and inform local police;
- Micro drones required a flight plan only in controlled airspace, while all operators were required to inform local police;
- Nano drones operated freely, restricted to 50 ft above ground in uncontrolled airspaces and enclosed premises [38].

c. Training Requirements

- Operators requiring a UAOP underwent a five-day training program covering regulations, flight principles, air traffic control procedures, weather, emergency handling, etc.

d. Constraints

- Drones were to fly within visual line of sight (VLOS) during the daytime;
- Photography using drones was permitted in well-lit enclosed premises with mandatory police notification.

e. No-Fly Zones

- DGCA designated 12 categories of “no-drone zones,” including a 5 km radius around high-traffic airports and 25 km from international borders [39];
- Additional no-fly zones included areas around strategic locations, state secretariat complexes, moving vehicles, ships, and aircraft.

The old policy involved many extra fees, a lot of paperwork, and numerous permissions to obtain. To address this issue and make drone flights easier and more accessible to everyone, a new policy was introduced in 2021.

#### 2.4.2. New Policy 2021

There were some shortcomings in the previous policy (2018). Since new developments happen each year in drone technology, policies and laws need regular updates to keep up. India revised its policy and introduced a new one in 2021. The key changes are listed below.

- Unmanned aircraft systems, including drones, can operate independently without human intervention;
- Before 2021, updated drone regulations required 25 separate submissions and a potential 72-step approval process, which has now been simplified to 5 forms and 4 stages;
- All previously issued authorizations, including unique identification numbers and certificates, have been revoked;
- Drone registration fees, previously based on size, have been standardized to INR 100 regardless of drone size [40];
- The required number of approvals dropped from 72 to just 4, and the number of forms went down from 25 to 5;
- The Civil Aviation Ministry is deploying a digital sky platform for centralized approvals that can be accessed on mobile devices;
- Drone flying zones are divided into yellow, green, and red areas, with reduced boundaries for flights near airports;
- Security clearances are no longer required for licensing and foreign ownership of drones;
- Companies are now allowed to be regulated by the Director General of Foreign Trade;
- The weight limit for permitted drones increased from 300 to 500 kg.

Moreover, the 2021 Drone Rules greatly reduced the regulatory and financial hurdles for drone adoption in Indian agriculture. With easier licensing procedures and the launch of the Digital Sky Platform, small and marginal farmers now find it simpler to legally access drone services [41,42]. Government initiatives like the Sub-Mission on Agricultural Mechanization (SMAM) provide subsidies ranging from 40% to 100% for buying drones, especially helping farmer producer organizations (FPOs) and self-help groups (SHGs). These changes have increased farm efficiency, cut input costs by up to 50%, and boosted yields by 15–25%. The policy has also promoted rural entrepreneurship through drone training programs, notably empowering women with initiatives such as NAMO Drone Didi [43].

### 2.5. Drone Adoption Trends Among Small-Scale vs. Large-Scale Farmers in India

Due to factors such as awareness, financial capability, and the extent of application, there are clear differences in how small- and large-scale farmers in India adopt drone technology.

A significant portion of small-scale farmers (73.33%) are becoming aware of the potential benefits of drone technology. Additionally, 61.67% of those who knew about drone technology reported using it in their farming activities, particularly for crop spraying. However, the high initial costs and technical complexities of operating drones present major barriers to wider adoption among this group. To address these challenges, initiatives such as Custom Hiring Centres (CHCs) have been established to provide drone rental services, making the technology more accessible and affordable [44].

Large-scale farmers, on the other hand, have higher drone adoption rates primarily because of their greater financial resources and ability to invest in advanced technologies. These farmers utilize drones not only for crop spraying but also for vegetation control, disease detection, and soil monitoring. Regional states such as Punjab, Haryana, Andhra Pradesh, and Tamil Nadu are leading in adopting drone technology [45]. Their capacity to purchase and operate drones allows them to increase productivity and improve efficiency across large farming areas.

To further illustrate the regional landscape of agricultural drone usage in India, the following Table 4 provides a comparative overview of drone adoption across various Indian states. It categorizes states based on their primary crops, policy support, market engagement, and overall adoption levels.

The table above highlights both the current situation and regional differences in agricultural drone use across India. Southern and western states, with stronger policies, institutions, and financial resources, are leading in adoption. Meanwhile, eastern and northeastern states continue to fall behind due to infrastructure issues, lower incomes, and fragmented landholdings. However, drone usage in India is expected to grow significantly in the future. The Indian drone market forecasts an 80% compound annual growth rate (CAGR) from 2020 to 2025 [68]. This growth will likely boost adoption rates in the regions that are currently lagging.

## 3. Methodology

To conduct a comprehensive literature review, as shown in Table 5, on the adoption of drones in Indian agriculture, Google Scholar was chosen as the main database for sourcing relevant research papers. It offers access to a wide range of peer-reviewed journal articles, conference proceedings, and technical reports from reputable sources such as IEEE Xplore, Springer, and other academic repositories. Additionally, online news articles, government publications and reports, blog posts, and annual market reports were analyzed to gain real-world insights into adoption trends, policy developments, and market dynamics.

A total of 366 sources were collected and organized using Zotero, a reference management tool. Among these, the distribution of document types was: 176 journal articles, 25 blog posts, 99 news articles from websites, 21 conference papers, and 6 book sections. These diverse references enabled a multidisciplinary understanding of the topic. Ultimately, 237 of these references were cited in the final review. Zotero facilitated efficient thematic tagging and classification of sources, which helped structure the literature around key themes.

The literature search was conducted using a combination of keywords and Boolean operators. The main search queries included terms such as “Drones in Indian agriculture,” “Agricultural drone technology India,” “Regulatory challenges for agricultural drones in India,” “Economic impact of drone adoption in Indian farming,” “Precision agriculture using drones in India,” and “Small-scale farmers and drone adoption in India.” Additional filters were applied to ensure relevance, including a publication year restriction to the last eight years (2018–2025). This ensured that only recent progress and emerging challenges in the field were considered.

Furthermore, only peer-reviewed journal articles, conference proceedings, and government reports were selected to ensure the reliability and credibility of the reviewed literature. Studies focusing on global drone applications were excluded unless they provided specific comparative insights relevant to India’s context.

The paper selection process consisted of several stages. First, titles and abstracts of the search results were screened to remove studies that were not directly related to the research scope. The remaining documents then underwent a full-text review, during which key insights were extracted, focusing on economic feasibility, regulatory challenges, and technological progress.

To enable organized analysis, the chosen papers and sources were grouped into four thematic categories.

- Technological advancements (97 sources);
- Economic feasibility (53 sources);
- Policy challenges (41 sources);
- Adoption barriers for small-scale farmers (46 sources).

This thematic framework enabled a systematic understanding of the factors influencing drone adoption in India’s agricultural sector.

Despite the thorough and comprehensive approach, some limitations were encountered. Restricted access to paywalled content prevented the inclusion of certain valuable studies. Additionally, while Google Scholar indexes many documents, it also includes non-peer-reviewed sources. As a result, manual verification of each source’s credibility was performed to maintain the review’s integrity.

## 4. Indian Agricultural Drone Landscape

The use of drones in India is growing. Drones are becoming more important in Indian agriculture for several reasons. Some of these are listed below.

### 4.1. Precision Farming

More than half of India’s workforce is employed in agriculture and related sectors, making it the second most populous country in the world. Despite having the second-largest crop production and the most fertile land globally, it falls behind in expanding its share of the economy. Innovative and advanced technologies are needed to address these issues. Here, precision farming provides farmers with many opportunities to cultivate higher-yielding crops suited to specific areas. Precision agriculture enables farmers to use inputs such as water, fertilizers, and pesticides more efficiently, reducing waste and environmental impact [69]. Modern farming techniques and AI present promising solutions for precision agriculture. Technologies like precision farming, biotechnology, genetics, and drones are just a few examples that can enhance sustainability, productivity, and efficiency. Drones equipped with advanced sensors and imaging systems allow farmers to monitor crop health, soil conditions, and irrigation needs in real time [70,71].

Although precision agriculture is a vital part of farming, it remains an advanced and costly technology that works very efficiently, which is a major obstacle to its wider affordability and adoption in developing countries like India [72].

### 4.2. Shortage of Labor

The agricultural industry in India faces challenges due to limited labor availability because of its heavy reliance on manual work for tasks such as plowing, sowing, and harvesting. Although manual labor has been a core part of Indian farming for generations, it can still be physically demanding and time-consuming, which limits the scale and efficiency of farming operations. The decline in agricultural labor is a complex issue caused by various factors, including migration from rural areas to cities, decreasing interest among young people in farming careers, insufficient access to advanced farming technologies, and the division of farmland ownership [73]. Agricultural work is seasonal, meaning jobs are available only for a few months each year. This prompts workers to seek more stable employment in other fields, such as electrician and plumbing roles. Furthermore, the agricultural workforce continues to shrink because of migration to nearby urban areas in pursuit of better educational and job opportunities. The shortage of workers is also worsened by international migration for overseas job prospects. Additionally, government programs like the Mahatma Gandhi National Rural Employment Guarantee Act (MNREGA) [18], intended to support rural workers, unintentionally increase labor costs for farmers, aggravating the worker shortage. The lower social status associated with agricultural jobs discourages many people from choosing careers in this field. Therefore, drones can play a vital role in addressing labor shortages. They can perform tasks such as crop monitoring and pesticide application more efficiently, reducing dependence on manual labor [74].

### 4.3. Time Saving

Drones provide significant time savings for Indian agriculture. They can spray fertilizers, pesticides, and other crop protection products much faster than traditional methods [75,76]. Conventional farming practices in India often rely on limited modern technology. For example, the continued use of backpack spray pumps is outdated and inefficient, as they are labor-intensive and offer limited coverage compared to modern alternatives like tractor-mounted sprayers and drones.

**Figure 3:**
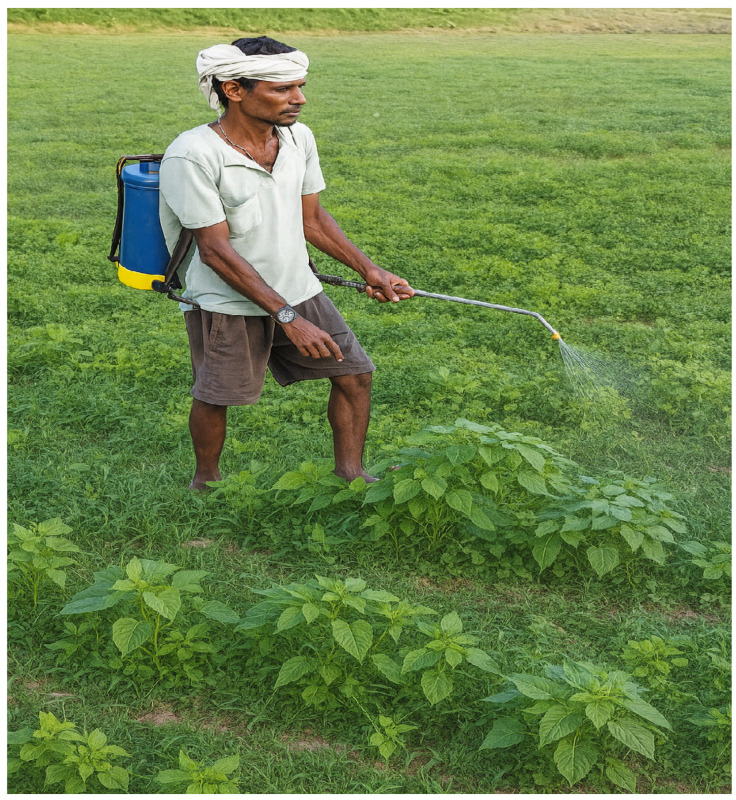
The farmer is using the traditional spraying method [77].

A farmer using a backpack spray pump to apply pesticides across a large field, as shown in Figure 3, may spend hours manually covering the area, which leads to inefficiency in time and labor [48]. This method also poses health risks to farmers due to direct exposure to chemicals. In contrast, a farmer using a tractor-mounted sprayer or drone can complete the task much faster with more precision, reducing labor needs and health hazards while ensuring even application and increasing productivity.

### 4.4. Effective for Pesticide Spraying

The excessive use of pesticides and fertilizers in Indian agriculture has raised concerns due to their negative effects on the environment, soil health, and human well-being [78]. Overapplication of these chemicals can cause water pollution, reduce biodiversity, lead to soil erosion, and pose health risks to farmers and consumers [79]. According to the Department of Agriculture and Farmer Welfare, India ranks among the top global producers and consumers of pesticides, with Uttar Pradesh and Maharashtra as the leading states for pesticide use. Multiple research reports from 2011 to 2020 have linked the presence of 45 different types of cancer among rural farmers in India to pesticide exposure [80]. Drone-based pesticide spraying greatly minimizes farmers’ risk of pesticide exposure. Research indicates that drone operators face lower risks of dermatitis, asthma, and chronic bronchitis compared to ground-based sprayers [81]. Moreover, drones use substantially less water for pesticide application—while traditional methods may require over 100 L, drones typically use only 5 to 6 L, an essential benefit in water-scarce areas. Using drones for pesticide application has also been shown to boost crop yields. For instance, a study in Haryana’s Kurukshetra District reported a 6.25% increase in yield and a 2.25% enhancement in crop quality [82].

### 4.5. Less Water Usage

**Figure 4:**
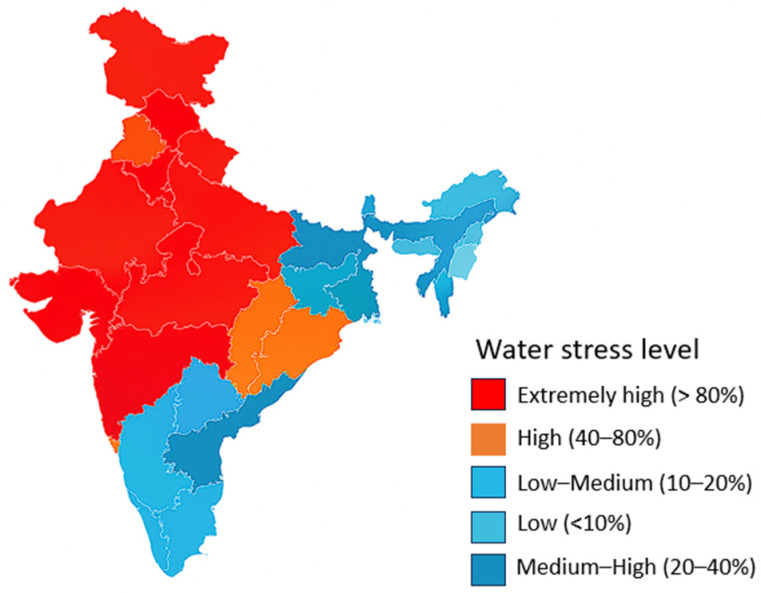
Water stress levels across Indian states, categorized from extremely high (>80%) to low (<10%) [83].

Drones are increasingly important for reducing water use in Indian agriculture, promoting more sustainable and efficient farming. It is important to note that water depletion remains a major challenge due to factors like groundwater over-extraction, inefficient irrigation, and climate change. Many regions experience water scarcity (Figure 4), impacting crop yields and farmers’ livelihoods. The excessive extraction of groundwater for irrigation, combined with poor water management practices, has caused a rapid decline in water tables nationwide. Climate change worsens this problem by causing unpredictable rainfall and extended droughts in many areas.

As a result, farmers encounter difficulties in securing water for their crops, leading to reduced agricultural output and economic struggles. Most irrigation relies on the overuse of groundwater in Punjab, Haryana, and Western Uttar Pradesh regions [84]. According to the Central Ground Water Authority’s 2020 Ground Water Estimation report, Punjab now extracts the highest amount of groundwater in the country. Irrigation uses up 97% of groundwater. Paddy is mainly cultivated through irrigation. Agriculture in Punjab is centered around paddy crops. The Punjab Agriculture Department reports that out of the total 3.59 million hectares, 3.13 million hectares were cultivated with paddy during the 2022–2023 kharif season [85]. Since excessive water use is a major issue for Indian farmers, drones play a critical role in addressing this challenge. Drones equipped with advanced sensors and imaging technology allow farmers to monitor soil moisture and crop health in real time. This facilitates targeted irrigation, ensuring water is applied only where and when needed, which greatly reduces overall water use. Currently, various water-saving methods such as mulching, crop rotation, and soil moisture management can be employed. When combined with drone technology, these practices significantly improve water efficiency in agriculture [86].

### 4.6. Current Use of Drone Applications in India

#### 4.6.1. Pesticide and Fertilizer Spraying

**Figure 5:**
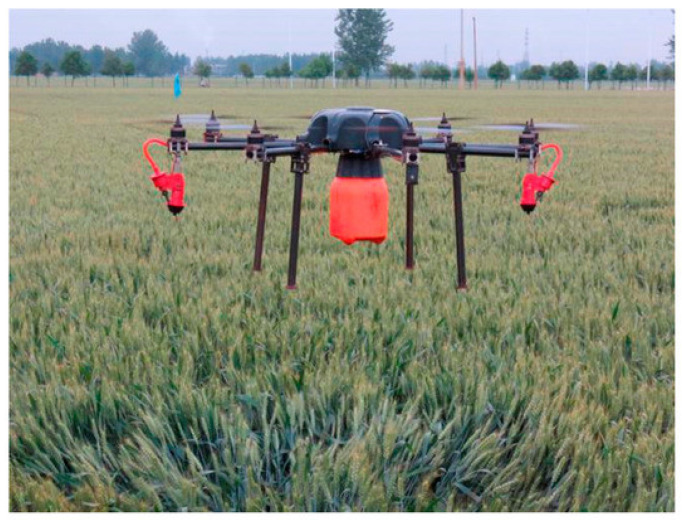
Pesticide spraying (retrieved from Wang et al. [96]).

In India, the adoption of drone technology in agriculture has driven significant improvements in crop management practices [87]. Drones are increasingly used for automated spraying of pesticides and fertilizers, effectively reducing health risks associated with manual application and boosting operational efficiency, as shown in Figure 5. The Indian Agriculture Ministry estimates that using a drone with a 10 kg payload costs roughly INR 350–450 per acre, based on six hours of daily operation covering about 30 acres [88]. Field data show that drone spraying provides uniform coverage, even for taller crops like sugarcane and orchards, leading to a 25–30% decrease in pesticide and herbicide use due to precise application. The technology also conserves water significantly, using droplets of around 50 µm compared to the 500 micron droplets in manual spraying, which results in an 80–90% reduction in water consumption. Additionally, drones can spray pesticides over 4000–6000 m^2^ of farmland in just 10 min, demonstrating their fast application ability [89].

These performance indicators are further supported by pilot studies and field experiments conducted across various Indian states. These studies offer empirical evidence of the operational and economic efficiency of agricultural drones, especially for pesticide application, crop monitoring, and input optimization. Table 6 summarizes key findings from recent drone trials across different crop types and regions in India, highlighting cost savings, labor reductions, and yield impacts.

#### 4.6.2. Crop Health Monitoring

Crop health monitoring with drones is rapidly growing in Indian agriculture, providing many advantages to farmers and the agricultural industry. Drones fitted with multispectral cameras, including near-infrared (NIR) and red-edge bands, allow for the calculation of vegetation indices like the Normalized Difference Vegetation Index (NDVI) and Enhanced Vegetation Index (EVI).

These indices offer valuable insights into plant health, chlorophyll levels, and water stress, enabling more precise and timely intervention [97]. There are various examples of practical crop monitoring using UAVs.

Adhao Asmita Sarangdhar and colleagues developed a system using UAVs to identify and manage diseases affecting cotton leaves. This system also tracks soil quality by connecting sensors to a Raspberry Pi, providing important data on moisture and other soil factors that influence crop growth [98].

Mohamed Kerkech et al. focused on detecting a specific disease (ESCA) affecting grapevines using UAV imagery and convolutional neural networks (CNNs). The UAV images were processed to classify the health status of the grapevines and create a disease map showing the potential for accurate crop monitoring and management [99].

Duana et al. used drones to monitor wheat growth with NDVI techniques. The UAV captured images using a multispectral camera and calculated NDVI values at different growth stages, which helped assess the health of the wheat crop and relate it to yield outcomes [100,101].

#### 4.6.3. Irrigation Management

The adoption of drone technology for irrigation is part of a broader trend in Indian agriculture toward more sustainable and efficient water management practices. This technology is likely to be used in areas where water conservation is a priority and where farmers have access to the necessary resources and support for implementing advanced agricultural technologies. As India faces increasing water scarcity due to climate change, drones are being used to adapt irrigation practices in affected regions. A case study in the Coimbatore District, Tamil Nadu, revealed that farmers have adopted drone technology for various agricultural practices, including irrigation management. Farmers have suggested that crops such as onions, cauliflower, and cabbage could be particularly well-suited for drone-based applications. Owing to their specific structural characteristics and growth patterns, these crops may benefit significantly from the precision and efficiency of agricultural drones. The use of drones for controlled irrigation ensures that crops receive the optimal amount of water, reducing the risk of overapplication, damage, and wastage [74].

Here, we specifically want to highlight the Indian farmers’ perceptions of drones. We have reviewed multiple studies across Tamil Nadu, Haryana, and other rural areas in India showing that drones significantly improve operations, especially in tasks like pesticide spraying, crop monitoring, and irrigation management (see Table 7). Farmers report that drones help lower labor and input costs, optimize chemical use, and conserve water resources. For example, Shankar et al. found that drone use resulted in a 30% reduction in costs and a 41% increase in income [92], while Noor and Noel documented a 6.25% rise in crop yield and a 2.25% improvement in crop quality [82]. Awareness of drone applications is also quite high, with one study indicating that 75% of farmers associate drones with spraying, 68% with pest and disease control, and 66% with irrigation by M. P P et al. [102]. Additionally, drones are viewed as promoting sustainable farming practices through more efficient resource use and less environmental impact. However, despite these positive perceptions, several barriers limit widespread adoption. Major obstacles include high initial costs, regulatory uncertainty, skill gaps, and technological challenges. Masih et al. reported that 70% of Indian farmers see cost as a key issue [103]. While the immediate environmental benefits of drones are well recognized, most studies overlook the long-term environmental impacts and issues related to disposing of drone equipment and batteries.

### 4.7. India-Based Drone Companies

India has experienced significant progress in drone development and adoption over the past few years. Many new drone startups have emerged to create agricultural drones that improve farming efficiency and precision. Numerous drone companies are actively involved in agricultural operations, and Table 8 outlines various Indian-made agricultural drones, including their manufacturers and key specifications. However, a few companies stand out in this field, such as Garuda Aerospace, which focuses on pesticide spraying and precision farming. Kisan Drones are equipped with artificial intelligence (AI), machine learning, and GPS sensors to give farmers real-time crop data. Dhaksha Unmanned Systems produces DH series drones known for their efficient spray systems and compatibility with farm management software. Additionally, Thanos Technologies developed the Syena series of agricultural drones, featuring intelligent spraying technology and real-time data monitoring. IdeaForge manufactured the AGRI series drones, recognized for their high payload capacity and advanced spraying systems suitable for large-scale farming [108].

Along with the drone models listed in Table 8, the Indian agricultural drone ecosystem includes a wide range of companies, startups, government agencies, and supporting organizations. These groups are not only developing and deploying drone technologies but also helping with training, market access, and research infrastructure. Table 9 highlights the main stakeholders driving the drone revolution in Indian agriculture.

Additionally, the following government and institutional bodies are crucial in promoting drone adoption in Indian agriculture.

- Directorate General of Civil Aviation (DGCA): oversees drone operations and certifies training organizations;
- Indian Council of Agricultural Research (ICAR): leads research projects on drone-based crop monitoring and pest control [109];
- State agricultural departments: conduct drone field demonstrations and support subsidies in states such as Gujarat, Himachal Pradesh, and West Bengal;
- Skill India Mission: creates certified training programs for drone pilots and rural youth [110];
- Farmer producer organizations (FPOs): promote drone service adoption and gather demand at the community level.

### 4.8. Key Statistics and Market Growth of the Indian Drone Market

**Figure 6:**
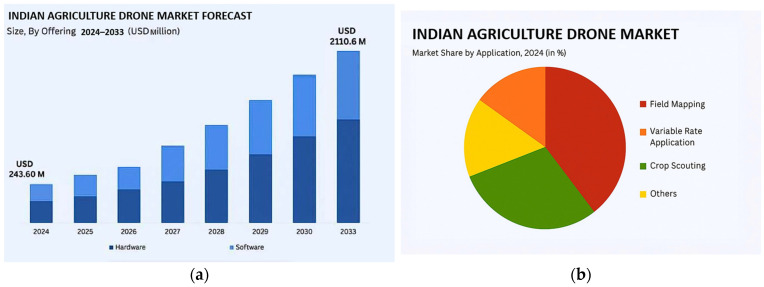
(**a**) Growth projection of India’s agricultural drone market (2024–2033); (**b**) Indian agriculture drone market share by application (2024).

The Indian agricultural drone market has experienced substantial growth, reaching a valuation of USD 243.60 million in 2024. According to projections by the IMARC Group, the market is expected to expand significantly, as shown in Figure 6a, reaching USD 2110.60 million by 2033, with a strong compound annual growth rate (CAGR) of 24.10% during the forecast period (2025–2033). This growth is mainly driven by robust government support, increasing adoption of precision agriculture, severe labor shortages, and ongoing technological advancements. Together, these factors make drone-based solutions more efficient, affordable, and accessible for Indian farmers [111]. In terms of application, field mapping represents the largest segment of drone use in Indian agriculture at 35.4%, followed by variable rate application at 24.6%, crop scouting at 18.2%, and other uses at 21.8%, as illustrated in Figure 6b.

The Indian government is promoting the use of drones in agriculture through various schemes and subsidies, such as the Production-Linked Incentive (PLI) scheme, which aims to boost the domestic manufacturing of drones and drone components in India. It was launched in September 2021 and provides financial incentives to companies involved in drone production, encourages investment, and reduces reliance on imports. The scheme allocates INR 120 crores over three financial years to double the combined turnover of all domestic drone manufacturers [112]. In Table 10, some PLI scheme beneficiaries are mentioned as the top drone manufacturers and importers across India. Additionally, government restrictions on foreign drone imports are encouraging domestic production and benefitting initiatives like the Kisan Drones program and state-specific drone policies in Himachal Pradesh, Gujarat, Goa, and West Bengal [113].

**Table 8:** Specifications of Indian agricultural drones.

Manufacturer	Drone Model	Tank Capacity (L)	Battery Life (min)	Flight Range (km)	Max Speed (m/s)	Ref.
Garuda	Garuda Kisan Drone	10 L	19 min	1.5 km	10 m/s	[114]
Nav Krishaak	NAV KRISHAAK	16 L	20 min	10 km	10 m/s	[115]
Dhaksha Drones	DH-AG-H1	12 L	35 min	0.5 km	5 m/s	[116]
Thanos	Syena-H10i	10 L	20 min	0.5 km	10 m/s	[117]
Prime UAV	Prime UAV	10 L	12 min	2 km	8 m/s	[118]
Labh Group	Labh Drone	10 L	17 min	0.5 km	10 m/s	[119]
IdeaForge	Q4I	10 L	40 min	4 km	7 m/s	[120]
Paras Aerospace	Paras Agricopter	10 L	20 min	N/A	4 m/s	[121]
Marut Drones	Agricopter AG 365	10 L	22 min	N/A	N/A	[122]

**Table 9:** Key players and startups in the Indian drone agriculture market.

Company/Startup	Location	Key Focus/Contribution	Ref
Garuda Aerospace	Chennai	Precision spraying, crop health monitoring, DGCA-certified training, large-scale farmer outreach	[123,124]
IoTechWorld Avigation	Gurugram	DGCA-approved Agribot drones for spraying, broadcasting, soil/crop health assessment	[125]
Throttle Aerospace	Bangalore	UAVs for land mapping, surveillance, inspection, disaster management in agriculture	[126]
Aarav Unmanned Systems	Bangalore	High-resolution imagery for crop health monitoring and yield estimation	[127]
FlytBase	Pune	Autonomous drone platforms for crop monitoring, data collection, smart farming solutions	[128]
Marut Drones	Hyderabad	Multi-utility agri drones, pesticide spraying, direct seeding, drone operator training	[129]
BharatRohan	Delhi, Lucknow, Hyderabad	Drone-based hyperspectral imaging, precision agri-advisory, FPO partnerships, traceability platforms	[130]
FlyLab Solutions	Nashik	DroneDekho platform, precision farming, micro-entrepreneur empowerment, water conservation	[131]
Vyomik Drones	Hyderabad	Crop spraying, field mapping, health analysis, fertility monitoring	[132]
Dhaksha Unmanned Systems	Chennai	Agricultural drones, drone services, technology solutions	[133]

The agricultural drone industry is seeing major investments, with startups focusing on creating drone solutions customized for farming. The Indian fintech market, which includes AgriTech, is valued at USD 31 billion and is projected to reach USD 84 billion by 2025, growing at a CAGR of 22% [134]. Companies like Marut Drones, Bharat Rohan, AVPL International, and Dhaksha Unmanned Systems are attracting funding to manufacture drones and offer drone-based services.

Indian investors are showing interest in the drone-as-a-service business model because the average farm size in India is about five acres, making it well-suited for drone technology, especially for surveying, crop health monitoring, and precision spraying. This also lets farmers rent drones for various other agricultural tasks. For example, investors such as Lok Capital see potential in the agricultural drone sector but admit that real progress might take 5–7 years [135]. However, since the market is still in its early stages, time is needed for farmer education and adoption.

That there are several notable partnerships and government funding initiatives supporting drone development in India’s agricultural sector [136]. The Kisan Drone scheme provides grants covering up to 100% of the cost of an agricultural drone (maximum INR 10 lakhs) for various agricultural institutions [137]. Additionally, the government offers a 50% subsidy (up to INR 5 lakhs) for agriculture graduates establishing custom hiring centers (CHCs), and farmers’ producer organizations (FPOs) can receive up to a 75% subsidy on drone costs for demonstration purposes. The Drone Didi Program (DDP), launched in 2023 to train women in acquiring and piloting drones, now supports 15,000 women’s self-help groups with financial assistance and loans for drone purchases [138].

Moreover, international collaborations like the India–Japan partnership help improve drone hardware manufacturing, R&D, and testing infrastructure, supporting sustainable market growth [139].

Recent advancements in agricultural drone technology have resulted in major industry collaborations to modernize farming practices in India. One key initiative is the partnership between Thanos Technologies and the Indian Farmers Fertilizer Cooperative, Ltd. (IFFCO), established through a memorandum of agreement (MoA). This collaboration aims to deploy over 500 drones for aerial fertilizer spraying, covering about 1 million acres of farmland across multiple states, including Telangana, Andhra Pradesh, Gujarat, Madhya Pradesh, and Tamil Nadu [140].

### 4.9. Success Stories in Indian Farms

Multiple case studies have been conducted on Indian farms that have benefited from drone technology.

In Punjab, farmers use drones for efficient pest control in cotton fields, resulting in less pesticide use and higher yields. Similarly, in Maharashtra’s vineyards, drones are used to spray fungicides, ensuring even coverage and lowering labor costs [141].

Darubrahma Automation Robotics, a startup founded by Rajendra Kumar Das, has revolutionized agriculture across multiple states in India. Since its inception, their drones have covered over 30,000 acres of farmland in states like Odisha [142].

In Tamil Nadu, farmers have adopted drones for spraying, sowing, and various agricultural activities. The state is known as the birthplace of precision agricultural drones, with farmers leading the way in adopting this technology. According to Agnishwar Jayaprakash, founder and CEO of Garuda Aerospace Pvt. Ltd., these drones have boosted productivity by 30% while reducing fertilizer, pesticide, and water costs by 70% [143].

**Table 10:** Drone manufacturers are beneficiaries of the PLI scheme.

Sr. No	Company	Headquarters	Year Established	Types of Drones	Key Models	Technical Specifications (Flight Range & Duration, Weight)	Technology Used	Primary Market	Revenue (2023)	Funding	Certifications	Ref.
1	Idea Forge	Mumbai, Maharashtra	2007	Surveillance, Mapping, Homeland Security, Agriculture	Q6 UAS, Q4i UAS, NETRA V3 + UAS	15 km & 120 min, Target Tracking	5G Technology, Live Streaming	Civil & Defence sector	INR 1860.1 million	330 cr.	DGCA & Bureau of Indian Standards (BIS) Certified	[144]
2	Raphe Mohib	Noida, Uttar Pradesh	2017	Surveillance drones, Logistic drones	MR-20, MR-10	20 kg Payload	Collective Intelligence, Ultra-Light Carbon Fiber Composites	Defense	270 cr.	132.55 cr.	DGCA Certified	[145,146]
3	AEREO (Aarav Unmanned Systems)	Bengaluru, Karnataka	2013	Surveillance & Land mapping	Aereo—ZFR, Aereo—INP	400 feet, 40 min, 13 kg payload	GIS (Geographic Information System)	Fields & Mining	N\A	15 million	DGCA Certified	[147,148]
4	Throttle Aerospace Systems	Bengaluru, Karnataka	2016	Delivery, Enterprise, Cargo	DOPO, TALV-TACT, NIMBLE-1	5 km, 55 min, 10 kg Payload	multispectral sensors, AES 256-bit encryption used	Defence & land survey	50 cr.	N\A	DGCA Certified, WPC Approved	[149,150]
5	Sagar Defence Engineering	Pune, Maharashtra	2015	Search & Rescue, Inspection & tracking	Spectre M, Spectre P	20 km, 5 kg payload, 60 min	IR (Infrared Camera), cloud connectivity	Defense & military tech	1.67 million	3.3 million	DGCA Certified	[151,152]
6	Roter Precision Instruments	Roorkee, Uttarakhand	1936	Surveying & security	Trinity F90+, Roter RC-08	7.5 km, 90 min, 5.3 kg payload	Anti-collision Strobe and Position Lights, Lidar, and advanced RGB Sensors	Area mapping	N\A	N\A	N\A	[153]

A study conducted in the Virudhunagar district of Tamil Nadu examined 60 farmers, including 30 drone users. The results showed that drones offer digital, informative, and accurate field management for crops like rice, cotton, and corn [154].

## 5. Issues and Challenges

Indian agriculture has great potential for improvement through drone technology, which can boost efficiency and traditional farming methods. According to Price Water Coopers (PwC) research, the infrastructure sector in India holds the highest potential value for drone-powered solutions at USD 45.2 billion [155]. However, the widespread adoption of drones in farming faces significant challenges, such as the following.

### 5.1. Technological Challenges

#### 5.1.1. Hardware Limitations

Low-cost hardware remains a major limitation for Indian drones. One of the main issues faced by UAVs is their limited operational time, mainly due to battery constraints. Drones typically fly for 20–60 min because they carry heavier loads [156]. This restricts ground coverage per battery charge and increases operational costs. Enlarging the battery is not practical, as it would impact the drone’s weight and maneuverability [157]. Additionally, the limited payload capacity of agricultural drones can reduce the effectiveness of pesticides and fertilizers during field spraying. With limited capacity, drones may not carry enough chemicals to cover large areas efficiently, leading to incomplete coverage and diminished spraying effectiveness [158]. A lower payload capacity often means more frequent refills, interruptions in spraying, and higher overall time and resource use. Furthermore, limited payload capacity also restricts the amount of equipment and sensors that can be transported during operations, which can limit the variety and quality of data collected, thereby affecting the overall efficiency and effectiveness of drones in agricultural tasks.

However, with limited payload capacity, drones might struggle to carry vital equipment such as high-resolution cameras, multispectral sensors, or extra batteries needed for longer flights. This can hinder the drone’s ability to gather detailed and accurate information, which is essential for decision-making in farming practices. Researchers are developing advanced algorithms for drones so they can select shorter paths to cover large areas more efficiently. For example, a study discussed the use of a smart algorithm called adaptive multi-start simulated annealing (AMS-SA) to plan optimal routes for drones during flight. Tests show that this algorithm outperforms existing ones and highlight the benefits of using multi-compartment drones, combining trucks and drones, swapping drone batteries, and considering how package load impacts battery usage [159].

#### 5.1.2. Import Dependencies

Most drone components, such as airframes, propellers, power sources (batteries/engines), and sensors, are imported from countries including China, Europe, and the USA [160].

– Approximately 25% of airframes are imported;
– Propellers have a 75% import rate;
– Power plants/batteries/engines also see about 75% of their components imported.

Notably, around 75% of electronic speed controls, servos, and sensor payloads (including DSLR cameras, lidar, and thermal sensors) are imported. This heavy reliance on imports impacts the domestic manufacturing ecosystem and the overall indigenization of the drone supply chain in India.

#### 5.1.3. Data Collection

Achieving high accuracy in data collection remains a challenge because accurate data depend on high-quality sensors. Since India mostly imports drone components, including sensors, from other countries, procurement costs stay high, increasing overall expenses for drone manufacturers and end-users. Additionally, proper infrastructure is essential for storing data. During flight, drones produce large amounts of data through sensors and imaging technologies. Analyzing and interpreting this data requires specialized knowledge and tools. Integrating drone data with existing farm management practices and decision-making processes can be difficult without adequate infrastructure and support systems.

Raw drone data are meaningless without analysis. Drones use various sensors (RGB, multispectral, and thermal) to collect data in different formats. Compatibility issues arise when integrating data from different sources. Farmers struggle to find software that can handle diverse data types and integrate smoothly with existing systems [161].

#### 5.1.4. Lack of Training Infrastructure

Despite growing interest in drone technology and various government initiatives, the lack of accessible and comprehensive training programs remains a major obstacle for Indian farmers, especially small and marginal farmers in rural areas [162]. Most organized drone training courses are located in urban centers, making them geographically inaccessible to rural communities. Additionally, awareness about drone technology and training options is limited. Even when such programs are available, many farmers face barriers due to low digital literacy and limited exposure to advanced technologies. A shortage of certified instructors and DGCA-accredited training centers further limits the reach and quality of drone education in remote areas.

Furthermore, training materials are often not available in local languages, which hinders participation by non-English-speaking farmers [163]. Practical, hands-on sessions are also limited, with many programs mainly focusing on theoretical or simulator-based instruction. High costs, travel requirements, and the opportunity cost of leaving farms for training further discourage smallholder participation. These limitations not only slow adoption rates but also increase the risks of improper drone use and missed opportunities for agricultural productivity improvements.

#### 5.1.5. Charging Infrastructure Constraints

A major yet underexplored barrier to drone adoption in Indian agriculture is the lack of dedicated charging infrastructure in rural areas [164]. Most villages do not have established drone charging stations, and existing solutions are mostly in the experimental or pilot phase. In regions where drone use is growing, unreliable electricity supply in rural areas further limits the feasibility of consistent drone operation, especially since frequent battery recharging is needed during field tasks. Although solar-powered and renewable energy-powered charging stations have been proposed and tested in limited pilot programs, scaling up faces challenges due to high installation costs, limited energy storage capacity, and reliance on favorable weather conditions [165].

To better understand the economic feasibility of deploying such infrastructure in India’s rural areas, Table 11 summarizes the estimated equipment and installation costs for different types of drones and comparable EV charging stations, based on available data and similar EV infrastructure costs.

As shown in Table 11, the cost of establishing drone charging stations in India varies significantly based on the system’s type and complexity. While basic battery hubs can be set up for less than INR 40,000, autonomous drone docking stations may cost over INR 30 lakh. Solar-powered stations offer a promising off-grid option for rural areas but are still cost-prohibitive for small-scale farmers due to high initial expenses and weather dependency [173,174].

Additionally, many components (e.g., batteries, solar panels, and inverters) are imported, which increases costs and logistical challenges [175]. Without targeted subsidies or rural deployment models, this infrastructure gap remains a major obstacle to the widespread adoption of drones in Indian agriculture.

### 5.2. Environmental Constraints

India’s diverse climate and terrain, ranging from the arid deserts of Rajasthan to the lush forests of the Western Ghats and the towering peaks of the Himalayas, present significant environmental challenges for drone operations [176]. The tropical climate in many parts of India, characterized by high humidity, can affect drone performance and sensor accuracy. Additionally, sudden weather changes are common in tropical regions and can disrupt drone flights and data collection. In some areas, strong winds, rain, and extreme temperatures can hinder drone operation and impact their effectiveness in tasks such as crop monitoring and spraying [177]. For instance, high wind speeds can destabilize drones during flight, affecting data accuracy. Rain can damage sensitive drone components, causing malfunctions. Extreme temperatures can reduce battery life and flight stability, lowering operational efficiency. A study [178] compared drone flyability by analyzing historical weather data—such as wind speed, temperature, and precipitation—against manufacturer thresholds using computer simulations. Weather-resistant drones have considerably higher flyability than standard drones, especially in warm and dry regions. Enhancing weather resistance thresholds for wind speed and precipitation can significantly improve drone flyability, particularly in major population centers. Drone operators must vigilantly monitor weather forecasts and conditions to ensure safe and effective operations.

Moreover, many regions covered with dense forests can disrupt drone signals and restrict visibility, making it difficult to gather accurate data and perform surveillance. Areas such as the Himalayas pose extreme challenges for drone operations due to their high altitudes and rugged terrain [179].

### 5.3. Economic Challenges

Cost is a major factor for farmers when considering investment in agricultural drones. The average annual income of a farmer in India is around INR 77,976 (about USD 1000), which limits their ability to invest in new technologies. Although drones have become much more accessible in recent years, there are still significant upfront costs to purchase and operate these devices in agriculture. An entry-level drone meant for small-scale farmers typically costs between INR 50,000 and INR 150,000, while more advanced drones with features like better imaging, longer flight times, and obstacle avoidance can cost up to INR 500,000 or more. This can be prohibitively expensive for many marginal farmers [180]. In addition to the initial purchase, there are ongoing expenses such as repairs, replacement parts, and battery replacements that add to the total cost of ownership.

Thus, farmers find it difficult to buy and use drones, especially small and marginal ones, because they have small, fragmented landholdings of less than one hectare, making it hard to justify the cost of drone technology. Lack of government subsidies and financing options is also a factor. Drone adoption requires a large initial investment and raises concerns about future costs, so farmers may hesitate to adopt drones [141].

The second reason relates to funding and investment. Agricultural laborers, who make up a large part of the farming community, have limited access to institutional credit facilities, forcing them to seek loans from non-institutional sources at high interest rates [181]. Despite government initiatives like Kisan Credit Cards (KCCs) and new banking products designed for farmers, many still face difficulties in getting loans due to bureaucratic hurdles, collateral requirements, and limited financial literacy. These barriers make it difficult for small-scale farmers to invest in advanced technologies such as agricultural drones [182].

Third, maintenance costs and training for drone operations further increase the financial burden on many farmers. Investment costs may include the price of drones as well as additional expenses. Furthermore, rapid advancements in drone technology can require frequent upgrades, adding to the overall investment. The cost of investment (CoI) can be analyzed using the formula:

CoI = IAC + TC + MC + UC + RF

where

- IAC (initial acquisition cost) represents the purchase price of the drone and necessary accessories;
- TC (training cost) includes expenses related to training personnel for drone operation and data analysis;
- MC (maintenance cost) covers ongoing costs for drone upkeep, repairs, and software updates;
- UC (upgrade cost) involves expenses for upgrading drone technology to stay current with advancements;
- RF (registration fee) involves the mandatory registration fee that drone operators must pay to register their drones with regulatory authorities legally.

These costs emphasize the financial pressure of adopting drones. However, analyzing the economic benefits and return on investment (ROI) helps determine if drone technology is ultimately a profitable choice for farmers. To better understand the ROI and cost-effectiveness of drone adoption, we provide a comparison in Table 12 between traditional farming practices and drone-supported operations on a 50 acre medium-sized farm.

For pesticide spraying, traditional manual methods cost about INR 15,000 per season in labor and take up to 5 days to finish, often leading to chemical overuse. In comparison, drone-based spraying cuts the operation time to just 4 h and reduces costs to around INR 3000 per season. Moreover, chemical use drops by 30–40% due to more precise application, without affecting the yield.

Similarly, for crop monitoring, traditional field scouting costs about INR 8000 per month, covers only around 20% of the field per day, and has detection accuracy between 40 and 60%. Drone surveys, costing INR 1500 per month, can cover 100% of the field within 2 h and achieve detection accuracy of 80–95%, enabling earlier intervention and better decision-making.

Based on these operational advantages, the estimated annual savings range from INR 120,000 to INR 180,000 for a 50-acre farm. Factoring in an average drone investment of INR 3–5 lakh (depending on features), the payback period is approximately 2–3 years. These results demonstrate the economic viability of drone adoption for medium-sized farms.

For smallholder farmers who cannot afford drone ownership, service-based models such as drone cooperatives, farmer producer organizations (FPOs), and government-subsidized operators can provide the same benefits without upfront costs.

Financial Outcomes

- Potential annual savings: INR 120,000–INR 180,000 per 50 acre farm
- Payback period for drone investment: 2–3 years.

### 5.4. Social Challenges

Many rural farmers have a deep-rooted connection to traditional farming practices passed down through generations. They may see the adoption of new technologies, such as drones, as a threat to their existing methods [184]. A lack of awareness and understanding of the benefits of drone technology in agriculture also causes resistance. Farmers might not fully understand the advantages drones provide, leading them to stick with familiar conventional approaches. One reason for this is that there are few training and educational programs available to teach farmers about these technologies.

Second, the lack of technical knowledge and training is a significant obstacle to adopting drone technology in Indian agriculture. Operating drones requires specialized skills that most farmers currently lack due to their age. The shift from traditional farming methods to UAVs will largely depend on the age of farmers, as older farmers are more likely to stick with conventional practices [17]. Flying a drone accurately over fields or crops involves more than just pilot skills; it requires proper training, expertise, and understanding of flight operations and maintenance. Additionally, data analysis and image stitching from drone footage need specialized tools and analytical skills that most marginalized farmers do not possess [185]. In India, especially in rural areas, there is a shortage of trained instructors and courses on drone technology and its agricultural applications. Farmers find it difficult to access the necessary training [186].

## 6. Technological and Policy Solutions and Case Studies

Possible solutions that provide social and economic benefits must be implemented to address the problems and challenges discussed in Section 4. To that end, some possible solutions are mentioned.

### 6.1. Technological Innovations

Traditional farming methods are being transformed by next-generation agricultural technologies focused on boosting sustainability, productivity, and efficiency. The use of advanced technologies like artificial intelligence (AI), machine learning (ML), drones, and the Internet of Things (IoT) has revolutionized agriculture. These tools help farmers tackle challenges such as unpredictable weather, resource constraints, and productivity issues [187].

#### 6.1.1. AI/ML Applications in Drone-Based Agriculture

As we enter the AI era, the integration of current devices with AI is growing. In agriculture, artificial intelligence and machine learning (AI and ML) are becoming transformative tools that can significantly boost revenue by reducing losses, enhancing decision-making, optimizing resource use, and managing risks related to agricultural losses [188]. Additionally, several key applications where AI and ML have had a major impact are highlighted.

The most important role of AI in agriculture is in crop disease detection. Machine learning algorithms analyze high-resolution images to identify signs of stress, disease, and nutrient deficiencies in plants [189]. Aerial photos taken by drones are used to train machine learning (ML) models with a convolutional neural network (CNN) [190]. These models accurately evaluate plant health and determine whether a crop is affected by disease, enabling AI-driven decision-making to recommend effective mitigation strategies [191].

For example, Mohanty et al. developed a deep learning-based disease detection system that uses the Plant Village dataset to identify 38 different plant diseases with 99.35% accuracy. The system, trained on over 54,000 field images, allows for real-time disease detection through smartphone cameras, drones, and IoT-based monitoring devices. In practical scenarios, if a tomato leaf is infected with late blight, an AI model can detect the disease early and suggest immediate actions [192].

The second-largest use of AI in drones is precision agriculture. Precision agriculture (PA) is a farming management approach that uses information technology and various data forms to optimize farming practices and boost farm efficiency. It involves advanced technologies like sensors, GPS, remote sensing, and data analytics to monitor and control field variability in crops and soil conditions [193]. The main aim of precision agriculture is to help farmers make better decisions about crop management, irrigation, fertilization, and pest control using real-time data [194].

The integration of AI with drones is transforming precision agriculture (PA) by enabling advanced data collection and analysis, which is crucial for understanding real-time field conditions. Using data from drone sensors and satellite images, AI algorithms help farmers continuously monitor their fields to quickly detect pests, diseases, and nutrient deficiencies, allowing for timely actions that protect crop yields [195]. Additionally, AI improves resource efficiency by calculating the ideal amounts of fertilizers, water, and pesticides needed for specific areas, reducing waste and environmental impact. Machine learning-based predictive analytics also enable farmers to forecast crop yields using historical and current drone-collected data, supporting better planning and resource management [196]. Furthermore, AI combines different data sources to give a comprehensive view of agricultural conditions and optimize processes like irrigation. Overall, deploying AI alongside drones in precision agriculture transforms traditional farming into real-time decision-making systems, promoting higher productivity, sustainable resource use, and improved farming practices [197].

#### 6.1.2. Integration of the Internet of Things (IoT) with Drones

IoT is the most revolutionary technology in modern farming. The basic concept of the Internet of Things in agriculture involves cameras, sensors, and other smart gadgets that gather data from every farm operation [198]. As IoT-enabled drones are equipped with sensors and high-resolution cameras, they can collect real-time data on crop health indicators, weather conditions, and pest infestation levels [199]. In IoT-driven precision farming, various sensors, such as those for soil moisture, temperature, and humidity, constantly monitor crop growth and environmental conditions, supporting data-driven decisions for better farming outcomes [200]. Several studies have demonstrated the positive impact of IoT integration with drones.

A comprehensive review published in early 2025, titled “Harvesting the Future: AI and IoT in Agriculture,” highlighted how integrating IoT with drones has transformed crop management and precision farming. The review stressed that these technologies allow for optimized resource use and increased crop yields through real-time monitoring and data-driven decision-making [201].

Another study focusing on IoT-based precision farming showed that using sensor networks and data collection methods, including drones, allowed for the simultaneous monitoring of soil moisture, temperature, humidity, and crop health [202]. This method has resulted in significant improvements in resource conservation and productivity.

A global study published in February 2025 further confirmed these findings, stating that smart farming technologies, including IoT sensors and drones, have greatly contributed to improving agricultural practices, reducing resource waste, and increasing efficiency [203]. The study highlighted that using precision farming techniques and real-time monitoring systems allows farmers to make data-driven decisions about irrigation, fertilization, and pest control, leading to better crop quality and higher yields.

Furthermore, IoT-based drones enable automated farm management by sending collected data to cloud platforms, where farmers can view real-time insights through mobile apps [204]. Additionally, 5G-enabled drones improve real-time data processing, supporting swarm drone operations for large-scale farming [205].

Precision agriculture is evolving due to the integration of IoT, drones, and AI, which makes farming smarter, more sustainable, and more efficient. Future advancements in edge computing, blockchain, and machine learning will enhance automated decision-making in agriculture 4.0 [206].

#### 6.1.3. Solar-Powered Drones for Cost-Efficiency

Solar-powered drones, also known as solar-powered unmanned aerial vehicles (SPUAVs), are emerging as a cost-effective and sustainable solution for agricultural use. A major challenge for drones is their limited endurance [207]. Currently, drones mainly run on batteries, which means they must return to the ground often to recharge, typically after only a few hours of operation. Therefore, the next step is to harness solar energy as a sustainable and efficient power source for drones [208]. By using solar energy as their main power source, these drones reduce their dependence on traditional fuels, resulting in lower operational costs and less environmental impact [209].

Integrating solar panels enables SPUAVs to generate electricity, reducing the need for frequent landings and battery replacements. This longer operational time is especially useful for applications like long-duration surveillance, environmental monitoring, and precision agriculture, where continuous data collection is essential [210]. Energy management strategies (EMS) are vital for controlling the output of solar cells and batteries and efficiently distributing power across various operational stages. Moreover, technological advances in solar cells, rechargeable batteries, and electric motors have progressively improved the efficiency and capabilities of these drones.

The extended operating hours and lower energy dependence of solar-powered drones make them very useful for different applications. In agriculture, for example, drones can continually monitor crop health, identify early signs of disease, and improve irrigation and fertilization plans [211].

Additionally, the maintenance costs of solar-powered drones are generally lower. Traditional battery-operated drones need regular battery replacements because lithium-ion batteries deteriorate over time and have a limited number of charge cycles. Solar-powered drones, however, have fewer moving parts and rely less on consumable components, which reduces both the frequency and cost of maintenance. The solar panels are durable and have a long lifespan, often outlasting other drone components [212].

Although the initial investment in solar-powered drones may be higher than that of traditional battery-operated drones, the long-term cost savings can be considerable. A study on solar-powered UAVs for precision agriculture found that these drones could lower operational costs by up to 30% compared to conventional drones, mainly due to reduced energy and maintenance expenses [213].

### 6.2. Policy Recommendations

The current regulatory landscape for agricultural drones in India is overseen by the Directorate General of Civil Aviation (DGCA). As outlined in the rules and regulations regarding drones in Section 2, there were previously many challenges for farmers, such as mandatory remote pilot licenses, strict operational restrictions, and complex approval processes, which often led to high compliance costs and limited adoption. Many farmers, especially small-scale operators, face bureaucratic hurdles and a lack of awareness about drone regulations. To address these issues, the Indian government took steps to reform drone policies and regulations in 2021. It introduced a fast-track approval system specifically for agricultural drones and eased licensing requirements for small-scale farmers [214,215]. There is a need to increase the number of licensing centers in rural areas and develop specific guidelines for agricultural drones.

Furthermore, to encourage the use of drone technology in Indian agriculture, the government has launched various financial aid programs to assist institutions, farmer organizations, and individual farmers. These programs aim to lessen the financial burden of purchasing drones.

#### 6.2.1. Institutional Financial Assistance

Institutions under the Indian Council of Agricultural Research (ICAR), Farm Machinery Training & Testing Institutes (FMTTIs), Krishi Vigyan Kendras (KVKs), State Agricultural Universities (SAUs), and other public sector agricultural organizations are eligible for 100% financial assistance to purchase drones, up to a maximum of INR 10 lakhs per drone. This support enables demonstrations of agricultural drone applications in farmers’ fields, promoting awareness and adoption at the grassroots level. Additionally, farmer producer organizations (FPOs) can receive grants covering up to 75% of the drone cost to conduct on-field demonstrations.

For institutions that choose to rent drones instead of purchasing them, a contingency expenditure of INR 6000 per hectare is allocated to cover operational costs when leasing from custom hiring centers (CHCs), hi-tech hubs, drone manufacturers, or startups. In contrast, institutions that own drones for demonstrations receive INR 3000 per hectare for operational expenses.

#### 6.2.2. Financial Support for Custom Hiring Centers (CHCs) and Entrepreneurs

To increase access to drone services for farmers, financial support is provided for establishing custom hiring centers (CHCs) under Cooperative Societies of Farmers, FPOs, and Rural Entrepreneurs. These CHCs receive 40% financial aid (up to INR 4 lakhs per drone) to offer affordable drone rental services to small and marginal farmers. Additionally, agricultural graduates establishing CHCs are eligible for 50% financial support (up to INR 5 lakhs per drone) to promote entrepreneurship in precision agriculture.

#### 6.2.3. Individual Farmer Subsidies

Small and marginal farmers, including those from Scheduled Castes (SCs) and Scheduled Tribes (STs), women farmers, and residents of northeastern states, are eligible for 50% financial assistance (up to INR 5 lakhs per drone). Other farmers can receive 40% support, with a maximum of INR 4 lakhs per drone for individual ownership.

#### 6.2.4. Government Investment and Subsidy Disbursement

As part of the Kisan Drone Promotion Initiative, the government allocated INR 129.19 crores to promote drone use in agriculture. This includes INR 52.50 crores assigned to ICAR for buying 300 Kisan Drones and holding demonstrations over 75,000 hectares through 100 KVKs, 75 ICAR institutions, and 25 SAUs. Additionally, financial support has been provided to various state governments for delivering over 240 Kisan Drones to farmers with subsidies and for setting up more than 1500 Kisan Drone CHCs to offer drone services at affordable prices [216].

### 6.3. Training and Awareness Program

The government has recognized the importance of providing skills and training in drone operation to help farmers adopt drone technology effectively. Initiatives like the program launched by Drone Destination and Indian Farmers Fertilizer Cooperative Limited (IFFCO), which offers drone pilot training exclusively for women as part of the “Lakhpati Didi Yojana,” demonstrate efforts to close the skills gap in drone operation [24]. The scheme supports modernizing Indian agriculture by lowering labor costs. Additionally, this shows that perceptions of working women among rural Indians are changing. According to a government poll conducted in 2023, 80% of rural Indian men and a little over 41% of rural Indian women are employed in the formal sector [25].

By equipping women with the skills and knowledge to operate drones in agriculture, these training programs not only empower women but also improve farmers’ overall ability to utilize drone technology for various farming activities. Additionally, these initiatives help develop a skilled workforce that can maximize the potential of drones in agriculture.

Furthermore, to make drones more accessible at lower costs and empower rural women, several self-help group (SHG) initiatives have been established, including the “Namo Drone Didi” scheme, which was launched in December 2023. The scheme aims to train and equip 15,000 women SHGs for the 2024 to 2025 period, enabling women to work as drone pilots in the field. This initiative not only encourages technological adoption in rural areas but also lowers operational and drone purchase costs because fully trained women drone pilots receive a 30 kg drone free from the government [217].

Along with new drone regulations in 2021, India’s Ministry of Civil Aviation launched a digital sky platform. The main goal was to train drone pilots for India’s growing industry. As of September 2024, over 10,000 type-certified commercial drones have been registered on this online system, simplifying drone registration, pilot certification, and licensing for commercial use. The platform is key in helping youth become trained drone pilots by offering a centralized certification system, connecting aspiring pilots with approved training organizations, and providing accessible information on airspace rules. Currently, there are more than 10,000 registered trained drone pilots in India. However, this number is expected to increase significantly, with officials projecting a need for 100,000 drone pilots in the coming years. The platform’s impact goes beyond registration and training, supporting the broader drone ecosystem in India, including government efforts to boost drone use in agriculture through subsidies.

To effectively address the skill barrier, the training curriculum typically combines theoretical knowledge, simulation exercises, and hands-on practical training. It includes regulatory compliance (such as DGCA regulations, drone registration, and pilot licensing), technical skills (drone components, flight planning, and simulator and actual flight training), agricultural applications (precision spraying, crop mapping, multispectral imaging, and data analysis), as well as safety protocols and record-keeping. Course durations range from short-term certifications of about 5 days to advanced programs lasting several weeks or months, sometimes incorporating mentorship and job placement support.

Training costs vary among providers and programs: Private DGCA-certified courses typically range from INR 60,000 to INR 100,000 per participant, while many government-supported and Skill India initiatives offer free or heavily subsidized training, especially for farmers, SHGs, and farmer producer organizations (FPOs). Access remains a challenge since certified training centers are mostly located in urban and agricultural hubs, although some states have set up mobile and district-level centers to reach rural areas, as shown in Table 13. Many courses are now available in regional languages and in blended online–offline formats to enhance inclusivity and accessibility.

Despite remaining challenges in cost and geographic coverage, these programs play a crucial role in developing a skilled drone pilot workforce in India, thereby encouraging wider adoption of drone technology in agriculture and supporting the government’s vision for digital and precision farming [218].

**Table 13:** Drone training providers in India.

Organization/Academy	Location	Notable Features/Curriculum	Typical Cost	Ref.
Drone verse	Pan-India	DGCA-certified, comprehensive agricultural curriculum	INR 60,000 (+GST)	[219]
Drone Academy of India	Multiple/online	100% placement, practical GIS/photogrammetry modules	N/A	[220]
Telangana Drone Academy	Hyderabad + rural	State-supported, hands-on and simulator training	Subsidized	[221]
Multiplex Drone	Pan-India	Simulator, hands-on, regulatory modules, log book	Moderate	[222]
Skill Digital India	Online	Free/low-cost “Kisan Drone Operator” course	Free	[223]
Garuda Aerospace, Drone Acharya, Eagletronics	Multiple	DGCA-approved, field mapping curriculum	N/A	[224,225]

### 6.4. Public–Private Partnership

With government support, possibly through public–private partnerships, a sustainable model should be established. Subsidies are available to promote drone technology adoption, with significant financial aid provided to various groups. Currently, several government policies and schemes support public–private partnerships (PPPs) in the development of agricultural drones.

The Drone Promotion and Use Policy-2025, approved by the Madhya Pradesh government, aims to increase drone usage in agriculture and establish the state as a hub for drone technology. The Sub-Mission on Agricultural Mechanization (SMAM) provides grants for purchasing agricultural drones, covering up to 100% of the cost for various institutions and offering reduced percentages for farmer organizations and custom hiring centers.

Additionally, the NaMo Drone Didi Scheme was launched for 2024–2026 with a budget of INR 1261 crores, aiming to provide drones to 15,000 selected women self-help groups for agricultural rental services and offering 80% financial assistance. Furthermore, the Production-Linked Incentive (PLI) scheme, initially introduced in September 2021, promotes drone manufacturing, component production, and software development. These policies and schemes have established a supportive environment for PPPs in agricultural drones.

**Figure 7:**
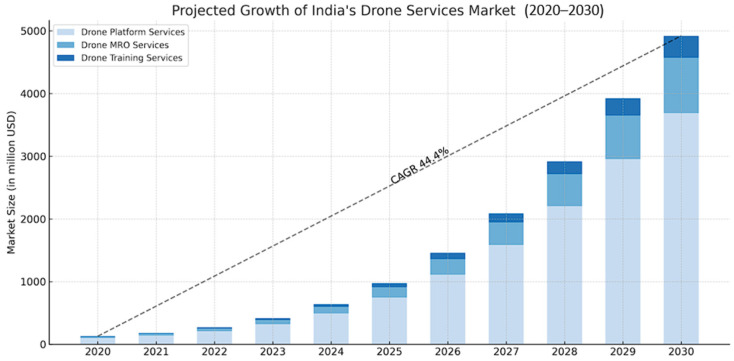
Projected growth of India’s drones-as-a-service (DaaS) market by segment (platform services, training, and MRO services) [226].

These drones-as-a-service (DaaS) models can enhance accessibility and lessen the financial burden on Indian farmers. They also include training, maintenance, and platform services. According to market forecasts, the DaaS sector is expected to grow from USD 130 million in 2020 to nearly USD 4.9 billion by 2030, with a compound annual growth rate (CAGR) of 44.4% (see Figure 7).

The above trend shows the growing feasibility of using service-based drones for smallholders, especially when paired with government initiatives like drone training and subsidies. It also highlights the expanding ecosystem around drone deployment, opening the door for broader agricultural transformation through service-oriented models.

In addition, several research institutions in India play a vital role in advancing drone technology through public–private partnerships (PPPs). For instance, the Institute of Infrastructure, Technology, Research, and Management (IITRAM) in Ahmedabad has established a Centre of Excellence for Drone Technology to promote cutting-edge research and education in unmanned aerial vehicles (UAVs) [227]. Likewise, the Vishnu Institute of Technology launched a drone center of excellence in 2018–2019 and has formed collaborations with private companies, such as New Propeller Technologies R&D Pvt. Ltd. and Airosspace R&D Pvt. Ltd. [228]. Universities also provide specialized education through undergraduate and postgraduate courses, as well as Ph.D. programs and certification courses in specific drone applications. They support industry collaboration by creating platforms for knowledge-sharing and building partnerships with private companies.

### 6.5. Research Solutions

In Table 14, some research-based solutions are listed that other authors have proposed to address these problems. These are the main concerns in drone implementation.

Qin et al. highlight the difficulties of providing internet access in rural areas and examine the potential of UAV-assisted networks powered by renewable energy charging stations. The proposed solution, backed by simulation results, indicates that this integration could be a practical way to address the limitations of UAV onboard battery life in rural regions. Future work and challenges in this field are also discussed [164].

Maddikunta et al. explore the potential of UAVs in smart farming, focusing on the importance of affordable and easy-to-operate control technologies. They propose using smart Bluetooth for UAV control and discuss the types of sensors used and the challenges faced. The study also highlights future roles for UAVs in agriculture, aiming to motivate farmers by addressing cost and control issues [229].

Kulbacki et al. examined how robotic process automation, image processing, pattern recognition, and machine learning can be combined with drones and satellites to improve precision agriculture. It emphasizes the advantages of high-quality remote sensing and the possibilities for variable-rate application, even though there is no unified legislation on drone use [230].

Parmaksiz and Cinar explore factors influencing farmers’ adoption of agricultural drones through face-to-face surveys and various analytical methods. The results emphasize the importance of government support, a preference for interest-free loans, and a greater willingness to rent rather than buy UAVs. These findings help identify farmers who are more likely to adopt UAV technology and offer valuable insights for decision-makers and market players to encourage its use in agriculture [231].

Majeed et al. examined the privacy and security issues with the Internet of Drones (IoD), emphasizing the vulnerabilities of small drone networks. They propose a new framework that uses intelligent machine learning models to improve data handling and privacy measures. This framework aims to protect drone networks from interception and intrusion, and its effectiveness has been confirmed with a benchmark dataset, showing strong results [232].

### 6.6. Case Studies

1. Cost Reduction and Entrepreneurial Impact of Drone Adoption in Rajasthan

This case study relies on publicly available reports from Indian news outlets and interviews with local stakeholders. It highlights the economic potential and scalability of drone use among tech-savvy young farmers.

In Hanumangarh district, Rajasthan, Ashish Beniwal, a 24-year-old engineering graduate, successfully adopted drone technology for agricultural purposes. After completing pilot training and obtaining the necessary license, he bought a 10 L agricultural spraying drone for INR 9.5 lakh in 2023.

He applied drone technology to crops like paddy, cotton, guar (cluster bean), and sugarcane, achieving the following significant reductions in input costs.

**Figure 8:**
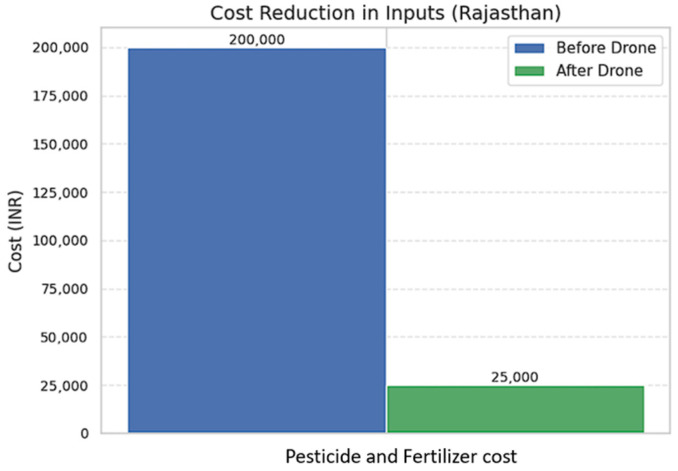
Input cost comparison before and after drone adoption in the Rajasthan case study.

- Pesticide and fertilizer costs for 30 bighas (approx. 7.5 acres) decreased from INR 2 lakh to INR 25,000, as shown in Figure 8;
- He reported a 40–50% decrease in chemical usage while maintaining crop yields.

Ashish also provided commercial drone services, covering 5000 bighas across Hanumangarh, Sriganganagar, and parts of Haryana, charging INR 200–250 per acre. His entrepreneurial effort not only created an additional revenue source but also encouraged fellow farmers, like Kuldeep Jalap, to start using drone services for their fields.

While Ashish’s experience highlights the economic viability and scalability of drone technology in Indian agriculture, such cases are currently limited to individuals with access to capital, technical skills, and formal training. However, with increasing government support, training programs, and targeted subsidies, the potential to replicate similar models across rural areas is expanding. Broader adoption will depend on expanding institutional support, improving affordability, and raising awareness among smallholder farmers [233].

2. Case Study: Adoption of Agricultural Drone Technology by a Woman Farmer in Punjab, India

This case is based on official coverage of the Drone Didi initiative, a government program launched in 2023 to train 15,000 rural women in drone operation. Jaswinder Kaur Dhaliwal’s story illustrates its transformative potential.

Jaswinder Kaur Dhaliwal, a 46-year-old woman from Rattian village in Moga, Punjab, was selected for training under the Drone Didi initiative, a government project aimed at training 15,000 women in agricultural drone applications [234]. Initially unfamiliar with drone equipment, she overcame her hesitation after receiving structured training in Gurgaon through the Indian Farmers Fertilizer Cooperative Limited (IFFCO) [235].

After completing her training, she started providing commercial drone-spraying services in June 2024. Her reported results include the following.

**Figure 9:**
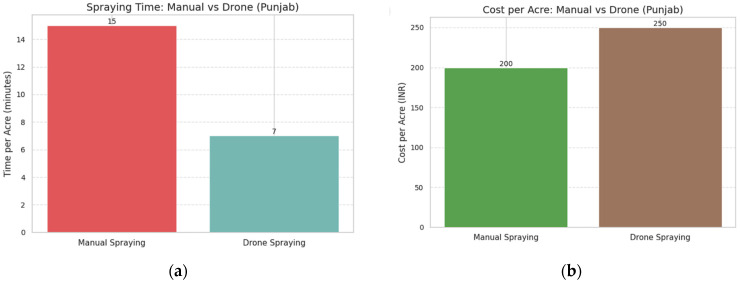
(**a**) Spraying time per acre: manual vs. drone method; (**b**) cost per acre for spraying: manual vs. drone method.

- Spraying over 200 acres of farmland at INR 250 per acre (see Figure 9b);
- Demonstrating efficiency improvements over manual spraying (usually INR 200 per acre);
- Reducing spraying time to 7 min per acre (see Figure 9a);
- Receiving positive farmer feedback on pesticide and fertilizer coverage.

To promote drone adoption, Jaswinder visited nearby farms and offered free one-acre demonstrations. In one case, this led to requests for drone spraying over 58 acres [236]. Beyond agriculture, she sees drone work as a way to address local social issues, especially among women. She said: “Drones are being used to supply drugs in Punjab, but I think only women can stop the drug menace by engaging themselves in such positive work” [237].

This case is not an isolated incident but reflects a growing national effort to include women in modern agriculture through drone technology. As part of the Drone Didi initiative, Jaswinder’s story demonstrates how organized training, affordable access, and targeted government programs can empower rural women and create entrepreneurial opportunities. While adoption is increasing, especially among self-help groups (SHGs) and farmer producer organizations (FPOs), broader success will depend on ongoing investment in infrastructure, affordability, and outreach that includes underserved regions.

## 7. Conclusions, Future Directions, and Open Issues

This study provided a thorough review of drone adoption in Indian agriculture, examining its advantages, challenges, and future prospects. It started by identifying the ongoing issues faced by Indian farmers, such as fragmented landholdings, low productivity, and limited access to modern technologies. In response, the paper assessed how drone technology—enhanced by AI, IoT, and precision tools—can revolutionize agricultural practices by increasing efficiency, lowering input costs, and promoting sustainability.

This review examined the Indian drone market’s expected growth in the coming year. Several domestic and international companies are emerging as major players in this field, contributing to hardware development, service delivery, and AI-driven analytics customized for agriculture.

This review also examined the regional mindset differences between small-scale and large-scale farmers regarding drone adoption. Although there is increasing interest and positive social perception toward drone use, challenges still exist, especially high initial costs, limited technical training, and insufficient infrastructure. These issues were analyzed from technological, economic, and social perspectives.

To overcome these barriers, the paper highlighted several solutions. These include policy recommendations such as institutional financial assistance, custom hiring centers, and individual subsidies. Additionally, public–private partnerships and service-based models like “drone-as-a-service” provide scalable, low-cost options for smallholder farmers. Training and awareness programs are also essential for building farmer capacity and encouraging long-term technology adoption.

Finally, this study highlights future directions and open challenges, stressing the importance of affordable models and better accessibility to promote the widespread use of drones in agriculture.

**Limitations:** This study was limited by its reliance on secondary data, which may not fully reflect real-time field challenges. However, this approach allows for a broad analysis of current trends, policies, and potential solutions, serving as a foundation for future empirical research. Although the study discusses policy recommendations, it may not fully address the practical challenges of implementing these policies at the ground level. Additionally, a more thorough examination of the potential long-term environmental impacts of widespread drone use in agriculture could improve the overall comprehensiveness of this study.

**Future directions:** Future research can explore autonomous drone technology for agriculture. Currently, a human operator is needed for drone operations. However, efforts are underway to enable drones to operate autonomously without human intervention, primarily using AI. The integration of AI and machine learning has greatly improved drone capabilities, allowing them to detect pests, assess crop health, and make data-driven decisions in real time [238]. An innovative advancement is the incorporation of generative AI technology, which provides sophisticated onboard processing for adaptive mission planning and better object recognition without depending on ground-based systems.

Additionally, swarm technology has gained popularity, enabling multiple drones to collaborate efficiently, expand coverage for large-scale operations, and support coordinated actions for complex agricultural tasks. Autonomous drones are becoming essential to precision agriculture, providing high-resolution imaging, real-time crop monitoring, and targeted resource application. They are equipped with advanced sensors, including multispectral and hyperspectral sensors for detailed plant health analysis and LiDAR technology for accurate 3D mapping [239].

Moreover, incorporating blockchain technology into drones is a key area of research. The combination of blockchain with drones is a quickly growing application, but it is not yet a mainstream or widely adopted industry practice. While promising pilot projects and research show their potential, most real-world deployments remain in the early stages [240]. Blockchain offers a strong security measure for drone operations. For example, drones generate and transmit sensitive data such as video, sensor readings, and location information, often over insecure wireless networks. Blockchain can provide a decentralized, tamper-proof ledger for storing these data, ensuring they cannot be altered or accessed without authorization. This is especially important for drones used in security, surveillance, and emergency response, where data integrity is crucial [241].

Additionally, Blockchain can assign unique cryptographic identities to individual drones, allowing only authorized users or systems to control them and access their data. This minimizes the risks of hijacking and unauthorized interference in drone missions, such as package delivery and surveillance flights [242].

While companies and researchers are actively exploring and testing these applications, blockchain-enabled drones are not yet common in commercial or government fleets. However, as drone use grows and the need for secure, autonomous, and scalable management increases, blockchain is expected to play a bigger role in supporting trusted, decentralized drone operations in logistics, defense, emergency response, and other sectors [243].

**Open issues: To complete our overview, we outline some unresolved questions and research challenges that need to be addressed for the smooth operation of drones.**

(1) Privacy concerns: Privacy is the biggest issue for drone flights. Cybersecurity for drone systems is crucial to prevent hacking, hijacking, and other cyber threats. There is a need to develop strong encryption protocols, secure communication channels, and tamper-proof hardware [244,245]. Researchers are working on advanced authentication methods to ensure that only authorized users can operate drones [246]. Additionally, research has focused on creating secure software update systems and using blockchain technology for safe data transmission and storage [247].
(2) Regulatory framework: Creating comprehensive and flexible regulations is crucial for overseeing drone operations across various sectors of agriculture. This includes establishing clear guidelines for drone registration, pilot certification, and operational limits. Ongoing efforts involve developing standards for drone identification and tracking systems to improve accountability and support effective law enforcement.
(3) Payload optimization: Enhancing payload capacity and efficiency while maintaining flight performance and safety is vital for broadening drone applications. This involves researching lightweight, high-strength materials and advanced structural designs to improve payload-to-weight ratios. Engineers are developing modular payload systems that enable quick and easy reconfiguration of drones for various missions [248].
(4) Human drone interaction: Creating intuitive interfaces and control systems for both professional operators and casual users is vital for widespread drone adoption. This includes designing user-friendly ground control stations with clear graphical interfaces and simplified flight controls. Ongoing efforts involve developing autonomous systems that can interpret high-level commands from users and convert them into complex flight maneuvers [249].
(5) Environmental impact: Evaluating the ecological effects of widespread drone use is essential for sustainable operation. This requires thorough studies on how drones affect wildlife, especially birds and flying insects. Researchers are creating quieter, more eco-friendly propulsion systems to reduce ecosystem disturbances [250]. Further research is necessary to examine the potential positive environmental uses of drones, such as wildlife conservation, pollution monitoring, and reforestation efforts [251].
(6) Open questions: Some additional remaining questions are as follows.
  - How to ensure smooth integration of drone technology with existing farm management practices and equipment;
  - How to handle concerns about data collection, storage, and sharing in drone-based agriculture;
  - Assessment of the long-term ecological impacts of widespread drone use in agriculture;
  - Establishing consistent international standards for agricultural drone operations;
  - Improvement in battery life and alternative power sources for extended drone operations.

These research areas and open issues offer opportunities for further progress in the field of agricultural drones, which could result in more efficient, sustainable, and productive farming practices in India and around the world.

## Figures and Tables

**Table 1:** Comparison of existing drone review articles.

Review Articles	Gupta et al. [15]	Katekar et al. [16]	Puppala et al. [17]	Ramanjaneyulu et al. [18]	Goyal et al. [5]	Our Review Article
Drones’ benefits	Yes	Yes	Yes	Yes	No	Yes
Economic and practical aspects mentioned (cost, training, job impact)	No	Yes	Yes	Yes	Partially	Yes
Challenges and limitations	No	Yes	Yes	Partially ^1^	Yes	Yes
Internet connectivity, infrastructure development	No	Yes	Yes	Partially	Yes	Yes
Case studies	No	Yes	Yes	Yes	Yes	Yes
Regulatory policies and laws	No	No	Yes	No	No	Yes
Research solutions mentioned	No	No	Yes	Yes	Yes	Yes
Drone categorizations in India	No	No	No	No	No	Yes

^1^ “Partially” signifies coverage of at least one but not all identified sub-factors: (1) Ramanjaneyulu et al. [18] covered cost savings (reduced spraying costs) and drudgery relief (reduced labor burden), but did not address training, job impacts, infrastructure, or environmental impact. (2) Goyal et al. [5] primarily addressed cost challenges, while training, job impact, and other practical aspects remain unexamined.

**Table 2:** Drone classification in India by weight.

Sr.	Types of Drones	Weights
1	Nano drones	Weighing up to 250 g
2	Micro drones	Weighing 0.25 kg to 2 kg
3	Small drones	Weighing 2 kg to 25 kg
4	Medium drones	Weighing 25 kg to 150 kg
5	Large drones	Weighing more than 150 kg

**Table 3:** Requirements for drone flying under the old Indian policy.

Category	Unique Identification Number (UIN)	Operator Permits	Estimated Approval Time	Height Allowed to Fly	Local Police Permission	Flight Plan and ADC
Nano drones	No	Yes	Not Required	50 feet	Yes	No
Microdrones	Yes	Yes	2–7 days	200 feet	Yes	No
Small drones and above	Yes	Yes	2–7 days	200–400 feet	Yes	Yes

**Table 4:** Regional trends in agricultural drone adoption across Indian states.

Indian State	Key Crops and Focus Areas	Notable Initiatives and Features	Drone Adoption Level	Ref.
Telangana	Paddy, cotton, pulses	Major pilot projects, research hubs, and 90% of national SOPs	Very high	[46]
Maharashtra	Sugarcane, cotton, horticulture	‘Namo Drone Didi’ scheme, rural SHG outreach, startup partnerships	Very high	[47]
Andhra Pradesh	Paddy, cotton, horticulture	Large-scale distribution to CHCs, early adopters	High	[48,49]
Punjab	Rice, wheat, sugarcane	Precision spraying, partnerships with manufacturers and FPOs	High	[50]
Haryana	Rice, wheat, sugarcane	Government incentives, precision spraying	High	[51]
Tamil Nadu	Rice, cotton	State-run programs, SOP contributions, research support	High	[52]
Uttar Pradesh	Sugarcane, wheat	Custom hiring centers, rising adoption in major crop belts	High	[53]
Madhya Pradesh	Wheat, soybean	Government projects, CHC-based adoption	Moderate-High	[54]
Karnataka	Ragi, maize, horticulture	State collaboration with manufacturers, farmer training	Moderate	[55]
Gujarat	Cotton, groundnut, horticulture	State pilot projects, growing private sector involvement	Moderate	[49,56]
Odisha	Paddy, pulses, horticulture	Bank loan support, pilot projects	Moderate	[57]
Rajasthan	Wheat, mustard, pulses	Kisan Drone centers, government subsidies	Moderate	[58]
Kerala	Spices, rubber, coconut	Pilot projects, limited but growing adoption	Low-Moderate	[59,60]
Chhattisgarh	Rice, pulses	Government-supported demonstrations, CHCs	Low-Moderate	[61,62]
Bihar	Rice, wheat, maize	Emerging adoption, government pilot programs	Low-Moderate	[63,64]
Uttarakhand	Wheat, rice, fruits	Demonstrations, training programs	Low	[65]
West Bengal	Rice, jute, vegetables	Pilot projects, limited drone use	Low	[66]
Assam and NE States	Rice, tea, horticulture	Early-stage, sporadic pilots, limited infrastructure	Very low	[67]

**Table 5:** Literature review criteria.

Aspects	Details
Database	Google Scholar
Time Range	2018–2025 (last 8 years)
Search Keywords	Drones in Indian agriculture, Agricultural drone technology India, Regulatory challenges for agricultural drones in India, Economic impact of drone adoption in Indian farming Precision agriculture using drones in India, Small-scale farmers and drone adoption in India
Inclusion Criteria	-Peer-reviewed journal articles -Conference proceedings -Government reports -Focus on the Indian agricultural sector
Selection Process	-Title and abstract screening -Full-text review -Thematic categorization
Thematic Categories	-Technological advancements -Economic feasibility -Policy challenges -Adoption barriers for small-scale farmers
Limitations	-Access to paywalled content -Manual verification of source credibility

**Table 6:** Summary of economic and operational efficiency outcomes from agricultural drone pilot studies in India.

Study (Year)	Location	Crop	Drone Applications	Cost Reduction	Yield/Income Impact	Operational Efficiency
Saranya et al., 2024 [90]	Pondicherry (TN)	Paddy	Fertilizer/pesticide spraying	6.04% per acre	Net income: INR 22,960 vs. INR 15,931 (non-drone)	Uniform spraying, improved input optimization
Y. A et al., 2024 [91]	Thanjavur and Madurai (TN)	Paddy	Precision input, crop monitoring	~17.5% total	Yield: 2032 vs. 1955 kg; net profit: + INR 7331	Improved application efficiency
Gowri Shankar R et al., 2024 [92]	Trichy and Pudukkottai (TN)	Paddy	Crop health monitoring, pesticide application	30% (cultivation); 12% (total)	Profit + INR 4355/acre; 41% income rise	Precision monitoring, targeted interventions
Dhivya C et al., 2024 [93]	Coimbatore (TN)	General Agriculture	Crop monitoring, resource management	Qualitative only	Yields optimized (no % data)	Optimized input application
Farheen Noor & Noel, 2023 [82]	Kurukshetra (HR)	Paddy	Pesticide spraying, health monitoring	Irrigation: 106.5 → 12.25; Labor: 200 → 50	Yield +6.25%; quality +2.25%	Water use: 5–6 L vs. 100+ L/acre; 5–7 vs. 35 min/acre
Chauhan et al., 2025 [94]	Pune (MH)	Tomato, okra	Decision support, irrigation/fertilizer optimization	Water ↓ 20–30%; fertilizer ↓ 15–25%	Tomato: +15–25%; okra: +10–20%; BCR: 2.5–3.0	High-res mapping; 80–85% pest/disease detection
Yallappa et al., 2017 [95]	Karnataka	Groundnut, paddy	Sprayer dev. and pesticide application	Spraying cost: INR 345–367/ha	N/A	Coverage: 1.08 ha/h; increased droplet uniformity

Note: Arrows (↑/↓) indicate increase or decrease, respectively, in the mentioned parameter. For example, “Water ↓ 20–30%” denotes a reduction of 20–30% in water usage.

**Table 7:** Summary of recent empirical studies assessing Indian farmers’ perceptions, adoption barriers, and application of agricultural drones.

Authors (Year)	Sample Size	Region	Drone Applications	Perceived Benefits	Main Barriers	Environmental Issues
Dhivya et al. (2024) [93]	N/A	Coimbatore, Tamil Nadu	Spraying, monitoring, irrigation	Improved efficiency, input optimization, labor saving	Cost, skill gaps, fragmented land, availability	Sustainability concerns
M. P P et al. (2024) [102]	60	Coimbatore, Tamil Nadu	Spraying (75%), pest control (68%), irrigation (66%)	Efficiency, high awareness, sustainability	Weather, maintenance, connectivity, policy, lack of training	Sustainable practices noted
Barathkumar et al. (2024) [104]	120	Coimbatore, Tamil Nadu	Spraying, monitoring, irrigation	Reduced chemical use, improved monitoring, higher yield	Cost, complexity, regulation	Water conservation concerns
Masih et al. (2025) [103]	40 (interview), 100 (survey)	India and Netherlands	Early pest detection, resource management	Sustainability noted by 60% (India)	Cost (70%), socio-cultural and policy barriers	Sustainability highlighted
Noor & Noel (2023) [82]	90	Kurukshetra, Haryana	Spraying, monitoring, irrigation, soil analysis	Yield ↑6.25%, quality ↑2.25%, labor and water savings	Awareness, cost, smallholder access, need for technical support	Manual spraying health issues mentioned
Sundar et al. (2023) [105]	N/A	Multiple districts, Tamil Nadu	Chemical spraying, crop protection	Efficiency, social/economic factors affect adoption	Cost, family influence, and policy barriers	N/A
Shankar et al. (2024) [92]	160 (60 UAV, 100 non-UAV)	Trichy and Pudukkottai, Tamil Nadu	Spraying, monitoring, pest control, irrigation	Cost ↓ 30%, income ↑ 41%, economic efficiency ↑ 90%	Cost, pilot shortage, maintenance, regulation	Mentioned runoff reduction, drift concern
Prabhu et al. (2021) [106]	N/A	India	Weed management, monitoring, resource optimization	Cost-effective, high accuracy (92.6–95.4%)	Training, flight time, small farms, low income	Health protection noted
Sangode (2024) [107]	N/A	India	N/A	N/A	Social anxiety, resistance, regulatory uncertainty, “environmental toll”	Environmental toll (vague)

**Table 11:** Estimated installation costs for drone charging infrastructure in India.

Type of Charging Station	Description	Estimated Equipment Cost (INR)	Estimated Installation Cost (INR)	Total Estimated Cost Range (INR)	Notes	Ref.
Basic Drone Battery Charging Hub	Small-scale battery chargers for drones (non-autonomous)	INR 1200–INR 9000	INR 10,000–INR 30,000	INR 11,200–INR 39,000	Suitable for small drone fleets, limited automation, and rural deployment	[166]
Autonomous Drone Docking Station	Weatherproof, automated drone docks (e.g., DJI Dock 2, M30)	INR 10,00,000–INR 25,00,000	INR 200,000–INR 500,000	INR 12,00,000–INR 30,00,000	High-end solution for continuous drone operations; requires stable power and connectivity	[167]
Solar-Powered Charging Station	Drone charging stations integrated with solar panels	INR 300,000–INR 800,000	INR 100,000–INR 300,000	INR 400,000–INR 11,00,000	Off-grid rural solution; cost varies with solar panel capacity and battery storage	[168]
EV Level 1 AC Charging Station	Slow charging, basic EV charger (analogous to drone charging)	INR 10,000–INR 20,000	INR 30,000–INR 50,000	INR 40,000–INR 70,000	Indicative of low-power drone charging setups	[169,170]
EV Level 2 AC Charging Station	Faster AC charging for EVs	INR 50,000–INR 100,000	INR 50,000–INR 2,00,000	INR 100,000–INR 300,000	Comparable to more robust drone charging hubs	[169,171]
EV DC Fast Charging Station	Rapid charging for EVs	INR 200,000–INR 500,000	INR 600,000–INR 13,00,000	INR 800,000–INR 18,00,000	High power, fast charging; upper bound for drone charging infrastructure	[172]

**Table 12:** Cost–benefit comparison for a medium farm (data adapted from [183]).

Aspect	Conventional Approach	Done-Based Approach
Pesticide Application		
Labor expense	INR 15,000 per season	INR 3000 per season (drone operation)
Time needed	5 days	4 h
Chemical usage	100%	60–70%
Crop Monitoring		
Labor expense	INR 8000 per month	INR 15,00 per month (drone surveys)
Coverage area	20% of field daily	100% of field in 2 h
Detection precision	40–60%	80–95%

**Table 14:** Solutions from different research scholars.

Ref	Title	Keywords	Problem	Method	Solution
[164]	Drone Charging Stations Deployment in Rural Areas for Better Wireless Coverage: Challenges and Solutions	5G, 6G Rural internet connectivity, Renewable energy, Network coverage,	Limited onboard battery and scarce electricity supply in rural areas	Used simulation, three practical scenarios	Proposes using renewable energy stations to enhance UAV network performance, supported by simulation results
[229]	Unmanned Aerial Vehicles in Smart Agriculture: Applications, Requirements, and Challenges	Smart farming, Bluetooth, Agricultural sensors, Cost	High costs and complexity in controlling UAVs could be barriers to adoption by farmers	Explores types of sensors suitable for smart farming.	Integration of Bluetooth smart-enabled sensors for farming applications
[230]	Survey of Drones for Agriculture Automation from Planting to Harvest	Robotic Process Automation, Image processing, Pattern recognition	Identifying the best applications of RPA and RS for maximum effect in agriculture	Analyzes the combination of RS technologies with UAS platforms for agricultural operations	Supports and develops a map or sensor-based variable rate application (VRA) using combined RS and UAS technologies
[231]	Technology Acceptance among Farmers: Examples of Agricultural Unmanned Aerial Vehicles	Farmer’s decision, Agricultural innovation	Limited information on the adoption of agricultural drones by farmers	Face-to-face surveys with 384 farmers	Government support Interest-free loans Renting over purchasing Cooperative help
[232]	Intelligent Cyber-Security System for IoT-Aided Drones Using Voting Classifier	Small drones, Cybersecurity, Privacy	Current small drone designs do not meet data transformation and privacy requirements for secure operation in civil and defense industries	Analyzes recent privacy and security trends Proposes a framework to enhance data transformation and privacy mechanisms in small drones	Employs intelligent machine learning models to enhance the security and adaptability of IoT-aided drones
